# In situ melt pool measurements for laser powder bed fusion using multi sensing and correlation analysis

**DOI:** 10.1038/s41598-022-18096-w

**Published:** 2022-08-12

**Authors:** Rongxuan Wang, David Garcia, Rakesh R. Kamath, Chaoran Dou, Xiaohan Ma, Bo Shen, Hahn Choo, Kamel Fezzaa, Hang Z. Yu, Zhenyu (James) Kong

**Affiliations:** 1 Grado Department of Industrial and Systems Engineering, Virginia Tech, Blacksburg, VA 24061 USA; 2 Department of Materials Science and Engineering, University of Tennessee, Knoxville, TN 37996 USA; 3 X-Ray Science Division, Advanced Photon Source, Argonne National Laboratory, Lemont, IL 60439 USA

**Keywords:** Mechanical engineering, Techniques and instrumentation

## Abstract

Laser powder bed fusion is a promising technology for local deposition and microstructure control, but it suffers from defects such as delamination and porosity due to the lack of understanding of melt pool dynamics. To study the fundamental behavior of the melt pool, both geometric and thermal sensing with high spatial and temporal resolutions are necessary. This work applies and integrates three advanced sensing technologies: synchrotron X-ray imaging, high-speed IR camera, and high-spatial-resolution IR camera to characterize the evolution of the melt pool shape, keyhole, vapor plume, and thermal evolution in Ti–6Al–4V and 410 stainless steel spot melt cases. Aside from presenting the sensing capability, this paper develops an effective algorithm for high-speed X-ray imaging data to identify melt pool geometries accurately. Preprocessing methods are also implemented for the IR data to estimate the emissivity value and extrapolate the saturated pixels. Quantifications on boundary velocities, melt pool dimensions, thermal gradients, and cooling rates are performed, enabling future comprehensive melt pool dynamics and microstructure analysis. The study discovers a strong correlation between the thermal and X-ray data, demonstrating the feasibility of using relatively cheap IR cameras to predict features that currently can only be captured using costly synchrotron X-ray imaging. Such correlation can be used for future thermal-based melt pool control and model validation.

## Introduction

Laser powder bed fusion (L-PBF) is a popular metal additive manufacturing (AM) method (also known as metal 3D printing)^1–4^. It uses a laser beam to form the part by repeatedly melting thin powders onto the substrate surface. Due to its layer-wise and location-specific deposition manner, L-PBF can create complex geometries that traditional manufacturing methods cannot. Furthermore, in L-PBF, site-specific material property control can be achieved by assigning site-specific printing parameters^5^. Given the above advantages, L-PBF has already been used in many industries such as medical, aerospace, and defense^6,7^.

However, L-PBF still suffers from large residual stress, deformation, delamination, and porosity^8–10^. The lack of understanding of the melting and solidification process under non-equilibrium conditions is the main barrier to achieving a high-quality deposition^11,12^. The melt pool and keyhole evolution must be well studied to enable local microstructure control and minimize defect formation^13^. More specifically, measurements of the thermal gradient and the solid–liquid boundary velocity are necessary.

The melt pool is the liquid-phase material bounded by the mobile solid–liquid boundary. The material properties of a 3D printed part are determined by the microstructure resulting from the thermal gradient and liquid to solid boundary velocity during the solidification process^14^. The keyhole is the vapor-depression zone in the center of the melt pool caused by overheating, vaporization, and the resulting vapor recoil pressure. The keyhole severity is critically related to the trapped gas porosity level during the melting and solidification process^15,16^. Note that lack of fusion and hot cracking can introduce additional porosity, but these issues are at a much larger scale that is typically considered unacceptable; therefore, they are not covered in this paper.

This work focuses primarily on single spot melting and solidification. While this method has limitations in applicability to traditional line scanning, it does offer a similar melt pool condition to random spot melting. The spot melt strategy is chosen in this paper because it is a novel method that is increasingly popular in metal AM as evidenced by recent (highly-cited) literature^17–21^. Furthermore, a comprehensive understanding of single spot melting is essential to build a platform for validating modeling efforts, which can then be extended to more complex problems. Experimental work by Dehoff et al*.*^17^ demonstrated that using the spot melt strategy can effectively manipulate the microstructural morphology from columnar to equiaxed.

Moreover, Raghavan et al*.*^18^ employed melt-pool simulations and a design-of-experiments approach to quantify the effect of critical process parameters on the equiaxed grain fraction in AM parts produced by spot-melting. Subsequently, Raghavan et al*.*^22^ used the aforementioned process-structure relationships to design a spot melt strategy to provide site-specific control of the build microstructure. A recent study by Kamath et al*.*^23^ showed that the spot melt strategy could produce a more homogeneous microstructure (crystallographic texture and phase fraction) than the raster melt. Additionally, Nandwana and Lee^19^ pointed out that depending on process conditions, the spot melt strategy can produce a larger melt pool than the raster melt strategy (thus, resulting in better remelting and bonding in the AM part).

The phase transformation of various materials has been studied in fusion welding^24–26^, but the small size (100–300 µm) and rapid liquid–solid phase change (m/s order of magnitude) of the melt pool during spot melting bring enormous challenges for in-situ measurements in L-PBF^7^. These L-PBF melt pool characteristics require the measurement to have both high spatial (< 10 µm/pix) and temporal resolution (> 10 kHz).

This work aims to: (1) Explore the feasibility of using synchronized advanced sensing methods (i.e., high-speed X-ray imaging, high-speed IR imaging, and high-spatial IR imaging) to observe the melt pool from both geometric and thermal perspectives. (2) Develop effective data preprocessing methods to handle the undesirables of raw data such as low contrast, high noise, and saturated pixels. (3) Extract critical physical properties of the melt pool under various processing conditions. (4) Establish the quantitative correlations between the thermal and X-ray data to enable single sensor-based quality control.

### Literature review

This section firstly summarizes metal AM quality sensing methods. Then, melt pool monitoring-related works are examined in detail from speed and spatial resolution perspectives. Following that, sensing signal and print quality correlation studies are reviewed. In the end, the research gaps are summarized, and the approach to address these limitations is introduced.

#### Metal AM-related sensing methods

Researchers have performed simulations, including finite element analysis and computational fluid dynamics, to study the fundamental physics of metal AM processes; however, due to the complexity of the L-PBF process, studies with reliable measurement are necessary^27–33^. Accordingly, numerous studies have been performed for L-PBF quality assurance with different sensors^34^. The measurements in AM processes include ex-situ and in-situ methods. Ex-situ sensors are not constrained by the size of sensors, and those are commonly used in AM include light (optical) microscopy (OM)^35^, transmission electron microscopy (TEM)^35^, and electron backscatter diffraction (EBSD)^36^. They can measure the microstructure of the built samples after production. Synchrotron^27^ and neutron diffraction^28^ are available to test the material's chemical composition and residual stress through the thickness. Porosity measurement can be achieved by CT and X-ray scans^37,38^, and surface topology can be acquired by 3D scanning and interferometry^39–41^.

In-situ sensors can achieve in-process quality monitoring and enable process control. They include thermal sensors (e.g., thermal couple, infrared camera, and infrared pyrometer^42–44^), optical sensors (e.g., CCD and CMOS camera, weld camera^45^, high-speed camera^43^), radiology sensors (e.g., high-speed X-ray imaging^46^), and acoustic/vibration sensors (e.g., microphone and fiber Bragg grating sensor^47^). More in-situ and ex-situ sensing methods are provided in McCann et al.’s review paper^48^, from which only limited methods can achieve the solid–liquid melt pool boundary velocity monitoring. Ex-situ methods offer many benefits for quality control and microstructure characterization but provide limited insight into the defect generation mechanisms. Among all the sensing methods, synchrotron X-ray imaging is the most powerful tool to achieve high spatial and temporal resolution during in-situ imaging.

#### Metal AM melt pool sensing-related works

Researchers have utilized synchrotron X-ray imaging to study the keyhole formation of L-PBF. For example, Martin et al.^46^ used synchrotron X-ray imaging on melt pool monitoring. They found that rapid formation and depression of the keyhole is a major source of porosity due to shielding gas and vapor metal trapped within the solidified component. Zhao et al.^7^, Parab et al.^49^, and Guo et al*.*^50^ used the high-speed X-ray imaging device available at the Advanced Photo Source (APS) Beam 32-ID-B in the Argonne National Lab, which has a 2 µm spatial resolution and 70 kHz frame rate. Various significant phenomena were captured, including vapor depression, melt-pool dynamics, and powder-spatter ejection. Ioannidou et al*.*^51^ summarized the world's synchrotron X-ray imaging L-PBF testing sites. The one at APS has the highest spatial and temporal resolution among all five locations.

However, only relying on X-ray imaging is not enough to fully understand the melt pool behavior because the thermal information such as cooling rate and thermal gradients is also essential. Kurz et al*.*^52^ found that the processing parameters significantly influence the thermal gradient and solid/liquid boundary velocities based on the physical model. Collins et al.^52^ discovered that changes in thermal input could lead to residual stresses, discontinuities, and unfavorable grain sizes and textures via changes in printing parameters. Bayle et al*.*^53^ combined high-speed infrared (IR) with a pyrometer and achieved 100 μm/pix spatial resolution on melt pool monitoring. Similarly, Chivel et al*.*^54^ used a similar setup and achieved 50 μm/pix spatial resolution. Heigel et al.^55^ achieved 36–52 μm/pix spatial resolution (the camera is tilted) on IR imaging through a viewport on an actual machine. Hooper^56^ used two high-speed co-axial cameras with 20 μm/pix spatial resolution to measure the cooling rate and boundary velocities of the melt pool surface. Qu et al*.*^57^ developed a hyperspectral imaging method to observe the melt pool, and estimated the temperature-dependent emissivity. These efforts in thermal monitoring are great attempts, but all of them lack dimensional melt pool information.

Wakai et al.^58^ combined the in-situ X-ray imaging with a pyrometer to monitor the melt pool of Ti–6Al–4V in L-PBF. However, their device's spatial and temporal resolutions (X-ray: 45 μm/pix with 500 Hz framerate, pyrometer: 13.3 μm/pix with 50 Hz framerate) were not high enough to observe rapid thermal and geometry changes. Gould et al.^59^ synchronized high-speed X-ray imaging with high-speed IR imaging to study L-PBF processes in real-time. They observed the phenomena during the melting and solidification processes such as vapor plums and spatter, quantified the thermal history and cooling rates, and demonstrated the concept of using synchronized high-speed X-ray and IR imaging to monitor the melt pool in L-PBF. However, their work did not investigate the effects of different printing conditions (laser power and dwell time), and had limited IR spatial resolution.

#### Metal AM data correlation

There is another motivation for the work accomplished in this paper. Currently, the in-situ melt pool geometry can only be obtained by high-speed X-ray imaging. However, it is impossible to implement the synchrotron X-ray source in an actual printer for quality control. Thus, there is a need to obtain printing quality information by a more accessible and cost-effective means, such as thermal imaging. Forien et al.^60^ used an in-situ pyrometer to correlate with the ex-situ X-ray measurement. They found that the pyrometer data has a great potential on porosity prediction. Mohr et al.^61^ combined an IR camera with a NIR high-speed camera to predict the lack of fusion voids found by ex-situ micro-CT. These two studies constructed very useful information. However, they still have limitations since the quality measures are ex-situ.

Seede et al.^62^ created a process parameter map to predict the porosity formation and microsegregation. This work is purely ex-situ, and the processing map’s resolution is low. Mondal et al*.*^63^ used machine learning-assisted modeling to predict the melt pool shape and calibrated the model by experimental trials. However, due to the high computational cost of the machine learning algorithms, this method is hard to achieve a real-time calculation with current technologies. Similarly, Liu et al.^64,65^ used a machine learning-based method to predict surface quality based on imaging data. Still, this method is relatively slow and only targets layer-wise prediction.

#### Research gap analysis

In summary, the L-PBF process requires in-situ process monitoring to understand the fundamental physics better. Due to the unique characteristic (miniature melt pool and rapid thermal cycle) of the L-PBF process, high-spatial and temporal geometrical and thermal monitoring is needed but seldomly reported in the literature. Additionally, since the high-speed in-situ geometrical information can only be achieved by high-cost synchrotron X-ray imaging, a relatively cheap sensing method (such as thermal imaging) is needed to predict them. The work reported in this paper synchronized the high-speed in-situ X-ray and high-speed IR camera to satisfy the > 10 kHz temporal resolution requirement and added a customized in-situ high-spatial IR camera to meet the < 10 µm spatial resolution requirement. This work discovers strong correlations between the high-speed X-ray and thermal imaging data, which have been observed with different printing conditions for Ti–6Al–4V and 410 Stainless Steel.

## Methods

To study the melt pool dynamics based on synchronized advanced monitoring techniques, namely, (1) high-speed X-ray imaging and (2) IR thermal imaging (both high-speed and high-spatial), the existing testing platform at the Advanced Photon Source (APS) was used and introduced in "Testing platform setup" Section. The preparation of the testing samples and the printing conditions of each experiment are discussed in "Sample preparation and experimental conditions" Section. The methods to preprocess the X-ray and IR data are presented in "X-ray data pre-processing and dimensions calculation methods" and "Thermal camera calibration, missing data interpretation, and thermal terms calculation methods" Sections, respectively.

### Testing platform setup

**Figure 1:**
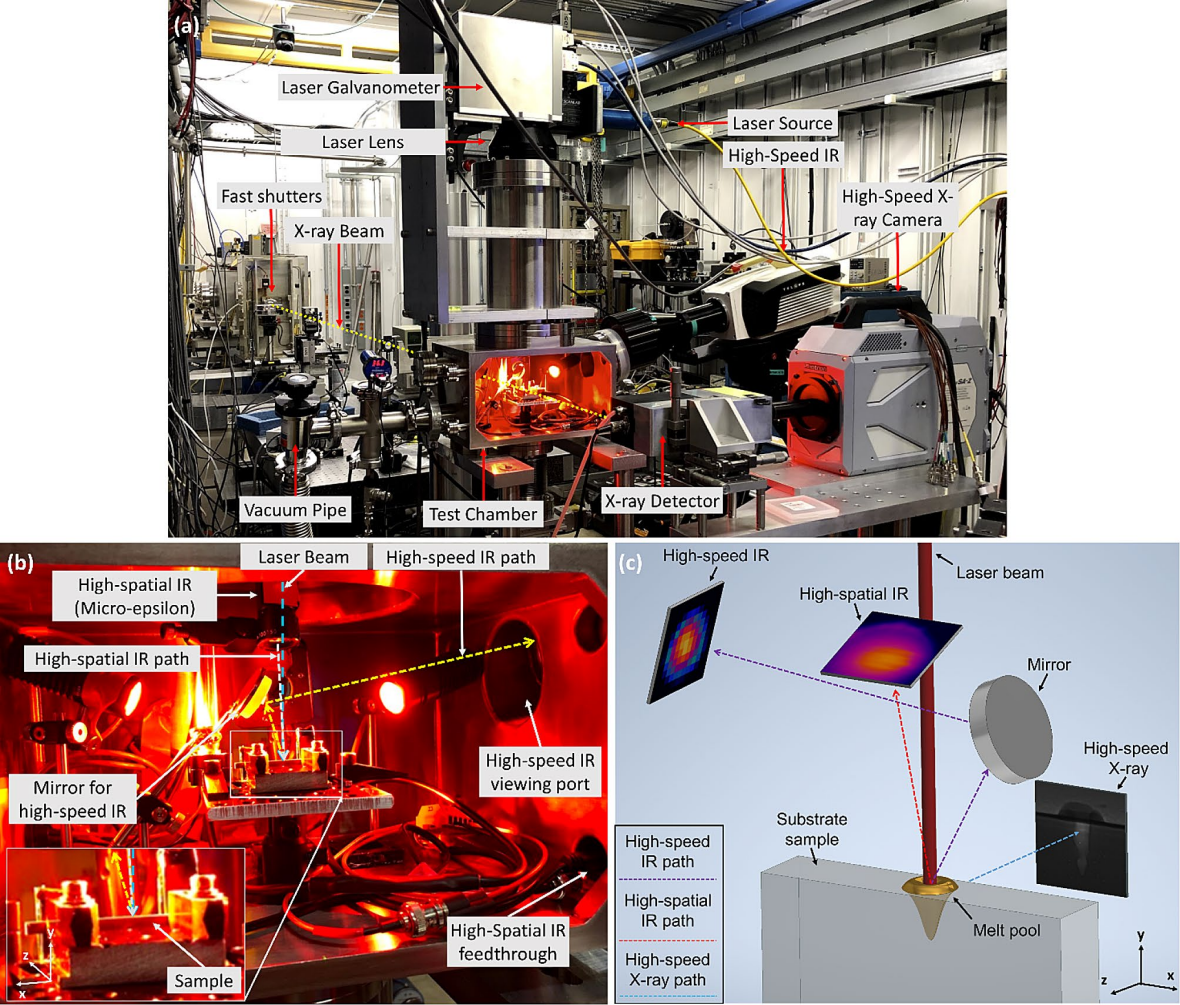
(**a**) The equipment layout of beamline 32-ID-B, APS, Argonne National Lab. (**b**) The layout of the testing chamber in beamline 32-ID-B, APS, Argonne National Lab that highlights the sensors used in this work. (**c**) The illustration of the viewing angle of high-speed X-ray, high-speed IR, and high-spatial IR. The aspect ratio has been enlarged.

The experiments in this study were performed at the 32-ID-B beamline of the APS, Argonne National Laboratory^66–69^. The APS synchrotron facility accelerates electrons to almost the speed of light. It uses the energy beams deflected by a magnetic field to perform experiments such as high-speed X-ray imaging. The beamline 32-ID-B was one of the testing sites in APS specialized for L-PBF research. The detailed schematic of the testing platform is illustrated in Fig. 1a. To investigate the L-PBF process, a Ytterbium fiber laser (IPG YLR-500-AC, USA) (1070 nm wavelength, Gaussian beam profile, measured M^2^ = 1.304, 520 W maximum power, and continuous wave) was used to create a melt pool on the sample (see Fig. 1a–c). This laser was redirected by a galvanometer (IPG FLC 30, USA) from the top of the chamber. The surface of the sample was positioned at 3 mm above the laser focal spot creating a beam size of 95 µm (1/e^2^ diameter). A beam of X-rays with a 24.4 keV first harmonic energy and a 0.508 Å corresponding wavelength was diverted from the synchrotron and entered the lab. This X-ray beam passed through the fast shutters, which were used to protect the imaging system by controlling the exposure time (500 ns in this work). The X-ray then entered the vacuumed test chamber, passed through the side of the sample, captured the melt pool, and illuminated the radiation detector (100 µm thickness LuAG: Ce scintillator). Such illumination was reflected by a 45° mirror, then captured by a 2 µm-spatial resolution high-speed camera at a 70 kHz frame rate (Photron FastCam SA-Z, USA).

As shown in Fig. 1a, b, in addition to the high-speed X-ray imaging, the melt pool was monitored by two IR cameras: the high-speed IR camera (Telops M3K, Canada) and the customized high-spatial IR camera (Micro-epsilon M1, Germany). The high-speed IR camera was equipped with a 1 × magnification lens which yields 30 µm spatial resolution and 1680 × 2160 µm field of view (FOV), and was calibrated to 2500* °*C by the manufacturer through black body tests (the emissivity calibration and measurement uncertainty will be provided in "Thermal camera calibration, missing data interpretation, and thermal terms calculation methods", and "Uncertainties" Sections, respectively)*.* The sensitive spectrum of this high-speed IR camera was 1.5–5.4 µm, and the sensing speed was 20 kHz. Due to its bulky size, the high-speed IR camera was positioned outside the chamber. It monitored the chamber through a Calcium Fluoride IR passing window (Lesker CF Flanged Exotic Lens Viewports, USA). The transmission spectrum of that window is provided in Appendix A1. As indicated in Fig. 1b, c a silver-coated mirror was adapted to reflect the top surface of the sample to the IR camera so that the high-speed IR camera could have a nearly perpendicular view of the melt pool surface.

A customized high-spatial IR camera was positioned in the laser duct to reveal more detailed melt pool surface information. The high-spatial IR camera was equipped with a customized 3X telecentric lens which yielded a 3.6 µm spatial resolution and a 260 × 195 µm FOV. It was calibrated to 1800 °C by the manufacturer through black body tests (the emissivity calibration and measurement uncertainty will be provided in "Thermal camera calibration, missing data interpretation, and thermal terms calculation methods" Section). It monitored the melt pool surface at a 1 kHz frame rate. So far, this customized setup has the highest spatial resolution among its peers. Since the laser wavelength (1070 nm) overlapped with the high-spatial IR’s sensitive spectrum (850–1100 nm), a 1064 nm blocking notch filter (Edmund Optics, USA) (12.5 mm Dia., OD 6.0) was added inside the telecentric lens to protect the camera sensor. The transmission diagram of the notch filter is provided in Appendix A2. The two IR cameras, the X-ray imaging, and the laser were synchronized by an analog trigger sent from a signal generator to ensure that all data collection started simultaneously.

### Sample preparation and experimental conditions

The samples used in this work were made of Ti–6Al–4V and 410 stainless steel (410 SS), respectively. These two materials were chosen because they are general alloys with broad applications. The Ti–6Al–4V samples were made from a 3.175 mm-thick, wrought Ti–6Al–4V sheet (Grade 5) purchased from TMS Titanium. The composition of the alloy as provided by the manufacturer is listed in Appendix A3. The sheet was cut using Wire electrical discharge machining (WEDM) into thinner rectangular sheets with dimensions of 3 mm (H) × 50 mm (L) × 0.7 mm (T). The rectangular sheets were then ground down to a final nominal thickness (T) of 500 µm (0.5 mm) with a surface finish corresponding to 600 grit sandpaper.

The 410 SS samples were manufactured using wire-fed laser direct energy deposition in the Manufacturing Demonstration Facility (MDF), Oak Ridge National Lab. The chemical composition of the feedstock is listed in Appendix A4. EDM was used to cut the build into rectangular sheets with dimensions of 3 mm (H) × 50 mm (L) × 0.5 mm (T). The rectangular sheets were then ground down to an approximate final thickness (T) of 300 µm (0.3 mm) with a surface finish corresponding to 600 grit sandpaper.

The experiments presented in this paper did not involve powder. Indeed, powder-on-plate studies best mimic the powder-bed fusion process. However, due to the complexity of powder experiments (variation in powder size, packing, oxidization, etc.), many repetitions are needed to obtain meaningful data with relatively low uncertainty. The scope of this work was to report the findings for a relatively large range of processing conditions and obtain a baseline for future powder-on-plate studies. Thereby, bare plates were used. Notice that the spattering will not significantly influence the result of this paper since the analysis is performed in a small area that only contains the melt pool (details provided in "Thermal camera calibration, missing data interpretation, and thermal terms calculation methods" Section).

The chamber was firstly vacuumed to 2.7 Pa and then backfilled with Argon gas until slightly over the ambient pressure. The detailed experimental conditions are provided in Table 1. The 410 SS trials have five printing conditions covering different melt pool conditions from conduction to strong keyhole. Each condition was repeated twice, one with the low-temperature IR filter and the other with the high-temperature IR filter (filter information provided in "Thermal camera calibration, missing data interpretation, and thermal terms calculation methods" Section). The Ti–6Al–4V trials included ten different printing conditions with the high-temperature IR filter. Trials #11-15 had the same energy input (in terms of a constant product of power by dwell time) and covered the melt pool mode from conduction to keyhole. Trials #16-20 were conducted to expand the processing conditions with different energy inputs.

**Table 1:** Experimental conditions for synchronized X-ray, high-speed IR camera, and high-spatial IR camera.

Trial number	Material	Laser power (W)	Dwell time (ms)	High-speed IR filter	With high-spatial IR?
1	410 SS	104	1	Low temperature	Yes
2	410 SS	104	1	High temperature	No
3	410 SS	130	2	High temperature	No
4	410 SS	130	2	Low temperature	Yes
5	410 SS	104	3	Low temperature	Yes
6	410 SS	104	3	High temperature	No
7	410 SS	260	1	High temperature	No
8	410 SS	260	1	Low temperature	Yes
9	410 SS	416	1	Low temperature	Yes
10	410 SS	416	1	High temperature	No
11	Ti–6Al–4V	104	2	High temperature	No
12	Ti–6Al–4V	416	0.5	High temperature	Yes
13	Ti–6Al–4V	208	1	High temperature	No
14	Ti–6Al–4V	156	1.3	High temperature	No
15	Ti–6Al–4V	130	1.6	High temperature	No
16	Ti–6Al–4V	312	1	High temperature	Yes
17	Ti–6Al–4V	130	0.6	High temperature	Yes
18	Ti–6Al–4V	156	0.8	High temperature	Yes
19	Ti–6Al–4V	260	0.8	High temperature	Yes
20	Ti–6Al–4V	156	2	High temperature	Yes

### X-ray data pre-processing and dimensions calculation methods

**Figure 2:**
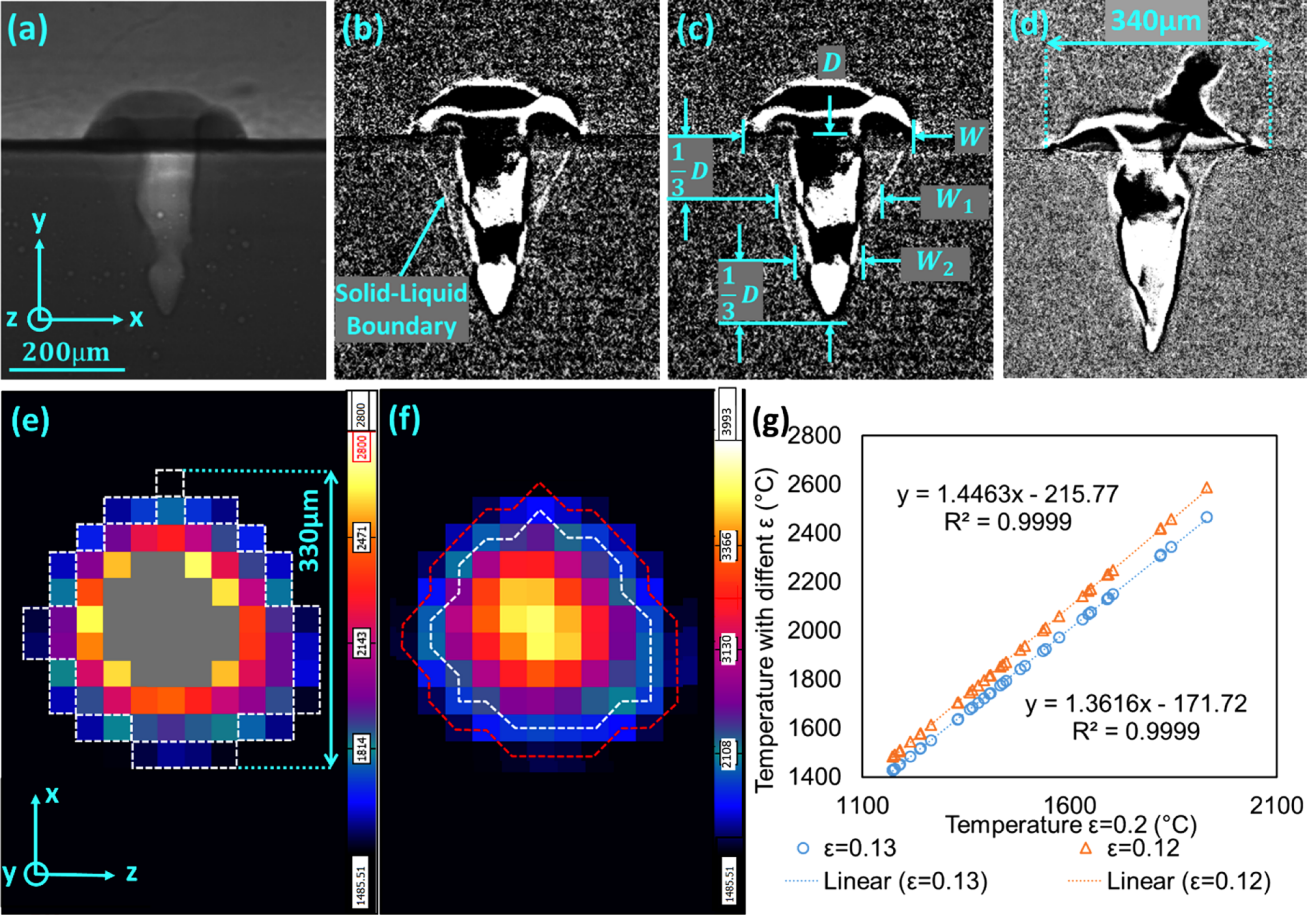
The (**a**) un-processed and (**b**) processed high-speed X-ray imaging of a 410 SS melt pool. The backgrounds have all been removed in the processed one, and the boundary of the melt pool becomes clear. (**c**) The definition of the melt pool dimension terms. (**d**) The processed synchronized high-speed X-ray image (Trial #12) at t = 0.4 ms (the first 1.1 ms is the laser delay time). (**e**) The high-speed IR image (*ε* = 0.12) of the melt pool at the same timestamp. The white dash region represents the pixels used for average solid–liquid boundary temperature calculation, and the grey-color region represents invalid readings due to temperature exceeding manufacture calibration temperature. (**f**) The corrected high-speed IR image (*ε* = 0.12) of the melt pool at the same timestamp. Notice the missing value problem has been solved. (**g**) The temperature conversion between different emissivity values. (Fig. 2d–g is discussed in "Thermal camera calibration, missing data interpretation, and thermal terms calculation methods" Section, and the temperatures in Fig. 2e and f are all in °C).

The X-ray imaging can capture the melt pool because the densities of the solid and liquid regions are different. However, this difference is so slight that it causes the images to have low contrasts. Additionally, the exposure time of the high-speed X-ray images is short (500 ns), which results in the low brightness of the images. These two problems make the melt pool challenging to observe in the raw X-ray images (see Fig. 2a). Therefore, a preprocessing technique was developed to enhance the image contrast. To enhance the boundary and remove the background, each pixel value in the *i-*th X-ray image is subtracted by the corresponding one in the (*i-j*)-th frame, where *j* is the lag between the two subtracting frames and can be determined by trying various values. This method will also mark the moving solid–liquid boundary by a thin line which essentially is the area of the melt pool changed in the time-lapse of *j* frames. The chosen value of *j* is a trade-off between the melt pool boundary clearness and the background removal effectiveness. In this work, *j* = 4 provided the optimal result. Afterward, the contrast of the images is enhanced by the histogram equalization method with 3% saturated pixels^70^. Figure 2b demonstrates that the processed melt pool is more apparent than the un-processed one in Fig. 2a.

After the melt pool boundary was enhanced, its shape was then characterized by the dimension terms: depth (*D*), width (*W*) (also refers as the melt pool diameter in this work), width at one-third of the depth (*W*_1_), and width at two-thirds of the depth (*W*_2_), illustrated in Fig. 2c. Zhao et al*.*^7^ also used *D* and *W* to characterize the melt pool. Our work uses additional width terms *W*_1_ and *W*_2_ to achieve a more accurate melt pool shape quantification. The melt pool dimensions were determined by directly measuring the melt pool in pixels and then scaling by the X-ray’s 2 µm spatial resolution. Our work also uses dimension ratios (the ratios between all the width terms against depth (*D*)) to further describe the melt pool shapes. They are *W/D, W*_1_*/D*, and *W*_2_*/D*. The velocities that melt pool depth and width changes are denoted as *V*_*D*_ and *V*_*W*_, respectively. These two values are calculated by the maximum *D* and *W/*2 (divided by two because the width shrinks from both sides of the melt pool) divided by the cooling time *t*_*c*_.

### Thermal camera calibration, missing data interpretation, and thermal terms calculation methods

As mentioned in "Testing platform setup" Section, two IR cameras, namely, high-speed IR and high-spatial IR, are used to monitor the melt pool surface. All the monitoring results and extracted values refer to the temperature at the surface. As for the high-speed IR camera, two different filters were used in the experiments. The low-temperature filter has a measuring range from 0 to 217 °C (used for observing the vapor), and the high-temperature filter ranges from 628 to 2500 °C (used for observing the melt pool). They have both been calibrated by the manufacturer via the black body tests. However, the emissivity (*ε*) still needs to be determined due to the variation in material, surface finish, measurement temperature range, and the blocking from the IR viewport. Taking Ti–6Al–4V as an example, the calibration procedure is presented as follows.

1. Find a synchronized X-ray and IR data set with a large melt pool. Then, locate the timestamp in both X-ray (Fig. 2d) and IR (Fig. 2e) data when the melt pool has a circular shape. Note that the melt pool surface may fluctuate, and a circular shape melt pool can provide more accurate emissivity calibration.
2. Estimate the diameter of the melt pool (*W*) by the method presented in "X-ray data pre-processing and dimensions calculation methods" Section. Then locate the corresponding pixels on the solid–liquid boundary in the IR data (white dash pixels in Fig. 2e) and calculate the average temperature.
3. Adjust the emissivity to make the average temperature of the boundary pixels (white dashed line in Fig. 2e) match the solidus temperature.

In this example, Trial #12 was used. At *ε* = 0.12, the average boundary temperature is 1640 °C, which is closely matched with the Ti–6Al–4V liquidus (liquidus temperature was used because the frame is during the melting rather than solidification) temperature reported in the literature 1655 °C^16^. This emissivity exactly matched the one presented in Gould et al*.*’s work^59^. Based on this method, the emissivity of 410 SS is estimated to be *ε* = 0.13. The liquidus temperature of 410 SS used for the calibration is 1450 °C^71^. The minimal reading of the IR cameras with the specified emissivity values is 1486* °C* and 1430 °C for Ti–6Al–4V and 410 SS, respectively. They are both below the melting point of these two materials.

As Fig. 2e shows, there are invalid pixels (colored in grey) in the center of the melt pool after the emissivity was adjusted to *ε* = 0.12. This problem is due to the presence of a keyhole, where the temperature exceeds the manufacturer's calibrated range. The emissivity difference between gas and liquid may also play a role. The values of these pixels can be estimated by the value of the same pixel with a higher emissivity value. To establish the extrapolation curve, all the readings of the pixels except the bad ones with *ε* = 0.12 and *ε* = 0.13 were paired with the reading at the sample pixel with *ε* = 0.2 and plotted in Fig. 2g. Since near-perfect linear correlations were observed, they were used to extrapolate the missing data. The processed version of Fig. 2e is shown in Fig. 2f, which does not have bad pixels. The data extrapolated might not accurately reflect the actual value because the emissivity is assumed to be constant for any location, which in reality will change depending on the temperature and phase. However, there is no such way to accurately measure the emissivity with extremely high temperatures (over 1727 °C). To the best of our knowledge, the direct measurement of liquid and gas emissivity of Ti–6Al–4V and 410-SS remains unknown^72^. Using a theoretical equation to extrapolate the emissivity is not accurate either because it assumes the surface roughness of the material is unchanged, which is not applicable when solid changes to liquid. The calibration and interpretation method proposed in this paper provides a baseline for estimation and is enough for in-situ control purposes.

As for the high-spatial IR camera calibration, the same method was applied to determine the emissivity value. Due to a customized 1070 nm notch filter added to the camera, each metal’s emissivity is smaller than the one used in the high-speed IR. They are *ε* = 0.04 for Ti–6Al–4V, and *ε* = 0.02 for 410 SS. The invalid pixel issue also occurs for the high-spatial IR camera because it was only calibrated to 1800 °C. The same method was used for extrapolating the missing value.

After the raw data has been corrected, feature extractions are performed. Unless otherwise specified, all thermal characteristics (e.g., *MPTEI* and *T*_*max*_) are based on high-speed IR observations with high-temperature calibration. The *MPTEI* (melt pool thermal energy index) is a term defined in this paper which has a strong capability to predict the melt pool geometrical information (which will be discussed in  "Correlation analysis and IR-based melt pool information prediction capability" Section). As the name implies, this term reflects the amount of thermal radiation from the melt pool due to the laser. To calculate it, the values of all the pixels are subtracted by the minimal reading (1486 °C) and then added together. This method effectively eliminates the influence of pixels that are not affected by the laser.

In addition, the maximum temperature of each frame is denoted as *T*_*max*_, and the average temperature of the melt pool surface in each frame is denoted as *T*_*avg*_. The frame just before the laser switches off typically has the maximum temperature of that trial. The *T*_*max*_ of that frame is denoted as *MaxT*_*max*_. The surface average cooling rate (denoted as *C*_*avg*_) is calculated by the *T*_*avg*_ after the laser shuts off and then divided by the time-lapses of one frame (0.05 ms). Similarly, the maximum surface cooling rate, denoted as *C*_*max*_, is calculated by the *T*_*max*_ change right after the laser turns off, then divided by 0.05 ms. The surface thermal gradient *G* is defined as the average thermal gradient at the surface solid–liquid boundary. As Fig. 2f shows, it is calculated by the difference between the average temperature of the pixels lying on the solid–liquid boundary (pixels on the red dash) and the average temperature of the next ring towards the center of the melt pool (pixels on the white dash), then divided by the high-speed IR spatial resolution (30 µm).

## Results and discussion

This section presents data examples obtained by high-speed X-ray, high-speed IR observations with high and low-temperature filters, and high-spatial IR. The discussion and interpretation of these results are provided in "High-speed X-ray observation", "High-speed IR low-temperature observation", and "High-speed IR high-temperature observation" Sections, respectively. The critical features of each trial, for example, boundary velocities and cooling rates, are reported in "High-spatial IR observation" Section. Additionally, the uncertainties of all the measurements and extracted values are discussed in "Uncertainties" Section.

### High-speed X-ray observation

**Figure 3:**
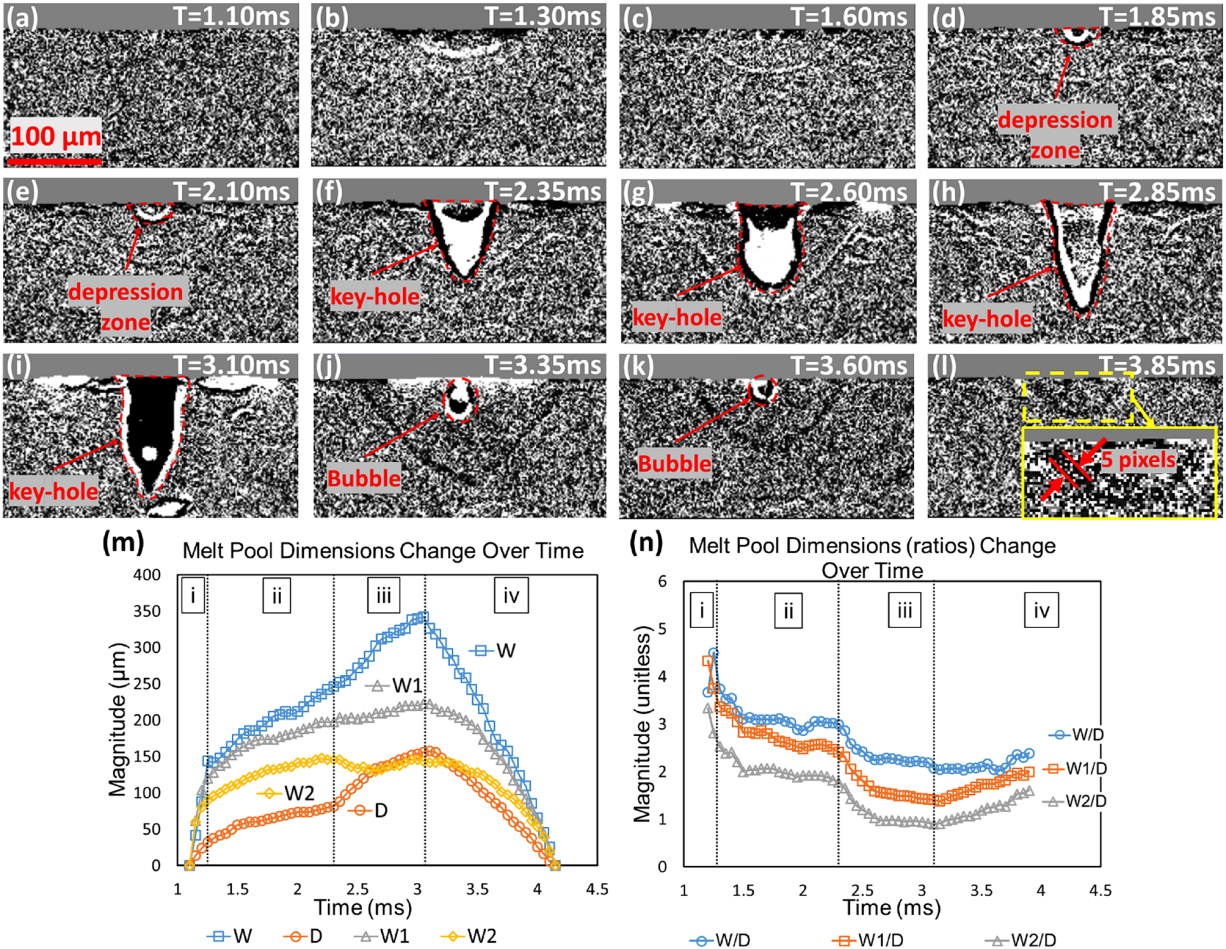
(**a**–**l**) The selected high-speed X-ray images of the melt pool of Trial #20. From the X-ray observations, phenomena such as keyholes and bubbles can be seen. (**m**) The melt pool dimensions change over time where blue squares, orange circles, gray triangles, and yellow diamonds represent the size of *W*, *D*, *W*_1_, and *W*_2_, respectively. Intervals i, ii, iii, and iv represent initial heating, conduction melting, keyhole melting, and cooling, respectively. (**n**) The melt pool dimension ratios change over time where blue circles, orange squares, and gray triangles represent the *W*/*D*, *W*_1_/*D*, and *W*_2_/*D*, respectively. Notice that *W* and *D* have a constant rate of change during the cooling process. The uncertainties of the extracted dimension values are ± 4 µm.

The high-speed X-ray imaging data can reveal the melt pool dynamics. Figure 3a–l show an example trial of high-speed X-ray imaging (Trial #20). Using the method presented in "X-ray data pre-processing and dimensions calculation methods" Section, the shape of the melt pool is obtained, and the dimensions are extracted. Figure 3m, n plot all the dimensions and dimension ratios along time. The entire process starts from *t* = 1.1 ms. It can be separated into four intervals: initial heating, conduction melting, keyhole melting, and cooling, represented by i, ii, iii, and iv, respectively. The initial heating interval is from *t* = 1.10–1.30 ms (Fig. 3a, b). The initial heating interval typically takes 0.2 ms. The occurrence of this interval is owing to the power rise time (0.05 ms, measured by the laser manufacturer) of the laser and the possible thin layer of oxidization on the substrate surface, knowing that the absorptivity of the 1064 nm laser of Titanium Oxide is less than 0.1 while the Ti–6Al–4V is over 60%^73,74^.

During the conduction melting interval (from *t* = 1.30–2.35 ms) (Figs. 3b–f), no keyhole except a small depression zone on the melt pool surface is observed. At this interval, the dimension ratios are large, representing the wide and shallow melt pool. From *t* = 2.35 ms (Fig. 3f), the keyhole starts to form, and the entire melt pool grows faster due to the increase in the irradiation absorption rate. This increase is due to the laser’s multiple reflections within the keyhole^75^. As shown in Fig. 3m, the melt pool depth has a growth rate change at *t* = 2.35 ms. This change corresponds to a transition from conduction to keyhole melting, so the dimension ratios drop rapidly (see Fig. 3n). This moment is also referred to as an inflection point^76^. The depth has a larger growth rate change than the width due to the keyhole.

Traditionally, the transition from conduction to keyhole melting is defined by aspect ratio (*W*/*D*) due to the lack of in-situ monitoring techniques. Goncal’s study^77^ showed that this is not an accurate way since there are cases that a wide melt pool contains a deep depression zone (see Fig. 3f). Benefiting by high-speed X-ray, Cunningham et al*.*^76^ defined the transition by input energy density. This method is still not complete enough because the melt pool development is not considered (Fig. 3a–l demonstrate that the keyhole could take as long as 1 ms to develop). In this research, the threshold is defined as when the keyhole depth exceeds 50% of the melt pool depth, which incorporates the consideration of the abovementioned scenarios.

When the keyhole forms, the depression zone instantaneously reaches the bottom of the melt pool. This phenomenon implies that the melt pool depth is the same as the keyhole depth during the keyhole interval. The cooling interval starts when the laser stops at *t* = 3.1 ms (Fig. 3i). All the dimensions begin to shrink. Figure 3j, k capture the motion of the bubble in this process, indicating the high possibility of porosity. These four intervals of dimension evolution could be considered piece-wise-linear, and such constant boundary velocities are observed in all trials.

The conduction and keyhole intervals do not always happen in the same trials. Trials #11, 14, 15, 17, and 18 have conduction melting intervals because the laser power is low or the melting time is short. Trials #12, 13, 16, and 19 do not have a clear conduction melting interval and directly enter the keyhole melting interval due to the high laser power. Note that Trial #16 has a few frames with oversized melt pools exceeding the field of view limit due to high laser power. Those frames were omitted for analysis.

### High-speed IR low-temperature observation

**Figure 4:**
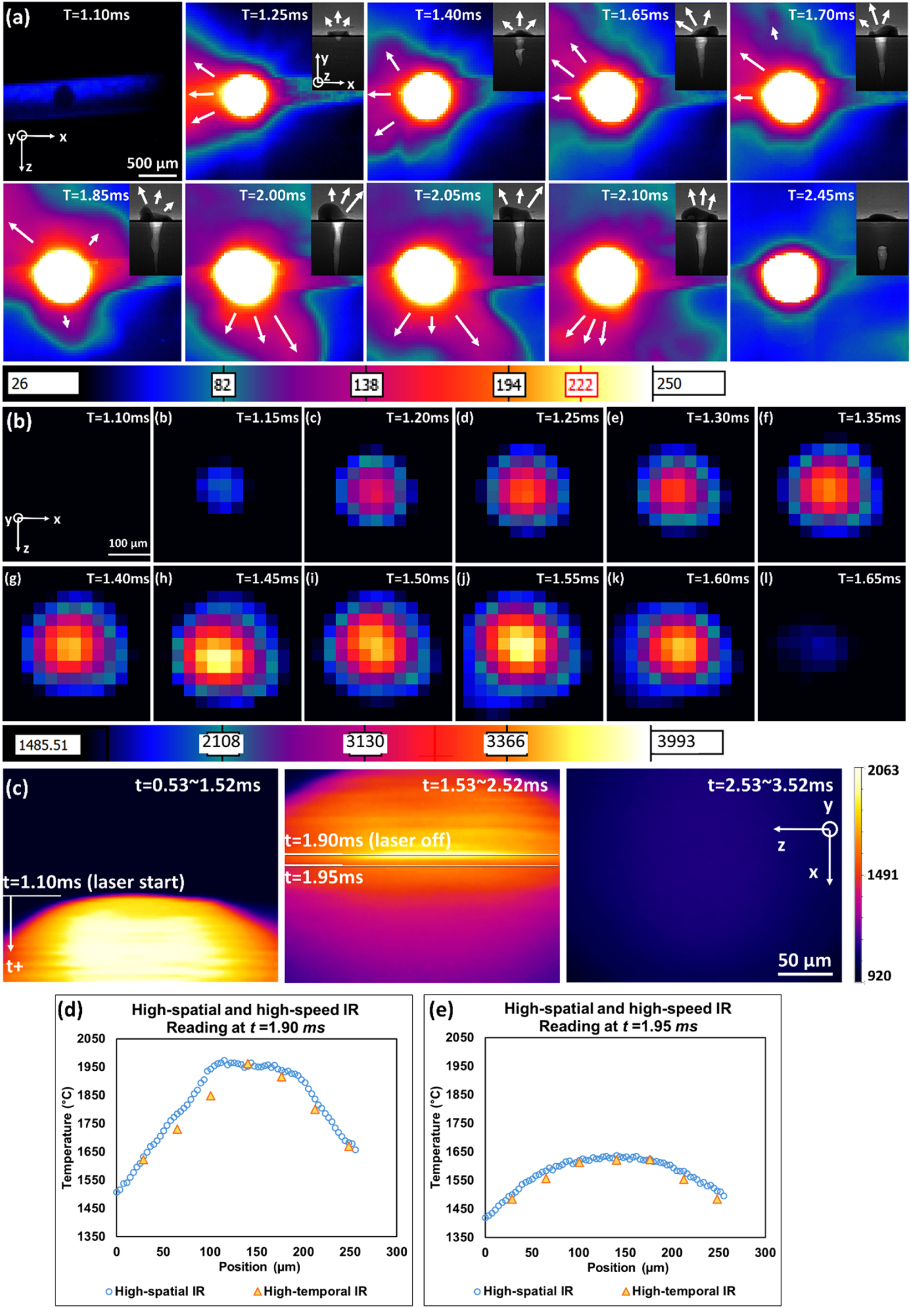
(**a**) Selected high-speed IR with a low-temperature filter showing vapor plume dynamics of Trial #9 (410 SS, 416 W 1 ms), where the dynamics of the vapor plume can be observed. The corresponding X-ray images in the upper right corner share the same scale as the IR images. (**b**) Selected high-speed IR with a high-temperature filter, showing melt pool temperature dynamics of Trial #12 (Ti–6Al–4V, 416 W, 0.5 ms). (**c**) The high-spatial IR observations of Trial #19 (Ti–6Al–4V, 260 W, 0.8 ms). The vertical direction contains both spatial and temporal information due to the camera sensor reading line by line from top to bottom. (**d**) The high-spatial and high-speed IR readings at the same location of *t* = 1.90 ms. (**e**) The high-spatial and high-speed IR readings at the same location of *t* = 1.95 ms. (Temperatures all in °C).

The high-speed IR low-temperature observation can reveal the motion and the intensity of the vapor plume. Figure 4a demonstrates an example of 410 SS vapor plume evolution (Trial #9). The corresponding X-ray images are provided in the top right corner and have the same scale as the thermal images. The white arrows in the images represent the vapor plume ejecting direction. Note the vapor has a negative x-direction drift in the IR image because the viewing angle is slightly tilted.

A consistency in intensity has been observed by comparing the X-ray data with the vapor plume. This part will be further discussed in "Keyhole prediction" Section. The comparison also reveals that the surface fluctuation of the melt pool determines the ejection direction of the vapor plume. Specifically, when the melt pool is relatively small and stable, the vapor intensity is mild and has a stable eject direction. In contrast, when the keyhole starts to form, the vapor plume intensity dramatically increases, and the ejection direction becomes unstable. This is because the surface fluctuates more intensively when the keyhole level is higher. The top part of the keyhole, combined with the surface liquid shape, can direct the vapor plume trajectory.

Figure 4a shows that the keyhole is shallow at *t* = 1.25 ms, and the vapor plume trajectory is narrow. Starting from t = 1.4 ms, the surface liquid starts to fluctuate and causes the vapor plume to become unstable. At *t* = 1.65 ms, liquid has been pushed to one side, directing the vapor plume to the other side. The next five frames show the vapor plume shifting to the opposite direction. At *t* = 2.1 ms, the plume oscillates back. After *t* = 2.10 ms, the vapor gradually stops because the laser is switched off.

### High-speed IR high-temperature observation

The high-speed IR with a high-temperature filter can capture the evolving temperature distribution of the melt pool. Such observations can be used to calculate thermal terms introduced in "Thermal camera calibration, missing data interpretation, and thermal terms calculation methods" Section. Figure 4b shows an example of such a dynamic temperature observation (Trial #12). From *t* = 1.15–1.60 ms, the melt pool growth can be observed. In this period, the hottest region is moving around, which indicates a volatile keyhole. The synchronized X-ray imaging has verified this insight because a strong keyhole was observed, and the melt pool surface is also shifting around. The last frame in Fig. 4b records the temperature distribution during the cooling. By comparing the last two frames in Fig. 4b, the *C*_*avg*_ and *C*_*max*_ were calculated based on the method mentioned in "Thermal camera calibration, missing data interpretation, and thermal terms calculation methods" Section. All the melt pool characteristics, including *MaxT*_*max*_, *G*, *C*_*max*_, *C*_*avg*_, *V*_*D,*_ and *V*_*W*_ are reported in Table 2. They are critical features that are useful for microstructure estimation. This table only contains Ti–6Al–4V trials since 410 SS trials mainly focus on keyhole-related testings. The measured maximum cooling rate has a range of 6.5–56 °C*/*µs, highly consistent with the value (1–40 °C/µs) reported in Hooper’s^56^ work. The thermal gradient presented in this work has a range from 1.6 to 8.7 °C/µm, which has the same magnitude as the number (5–20 °C/µm) presented in Hooper’s work as well^56^. Notice that the level of magnitude accuracy for microstructure estimation is usually enough^78^.

**Table 2:** Extracted thermal and geometrical characteristics for each trial.

Trial number	MaxT_max_ (°C)	G (°C/µm)	C_max_ (°C/µs)	C_avg_ (°C/µs)	V_D_ (m/s)	V_W_ (m/s)
11	1705	2.8	6.5	2.1	0.165	0.250
12	3561	8.7	56	11.0	0.356	0.163
13	2973	6.4	42	10.3	0.214	0.179
14	1990	5.3	13	4.4	0.177	0.255
15	1802	3.7	9.3	3.0	0.144	0.262
16	3594	7.5	54	9.7	0.266	0.137
17	1736	3.3	7.4	2.0	0.177	0.347
18	1843	1.6	8.1	2.4	0.179	0.300
19	3387	7.8	35	9.1	0.249	0.166
20	2964	6.6	39	7.8	0.157	0.172

The thermal-related term is based on data with a spatial resolution of 30 µm and temporal resolution of 0.05 ms. The boundary velocities are calculated based on data with a spatial resolution of 2 µm and temporal resolution of 0.0143 ms. All the uncertainties are presented in "Uncertainties" Section

### High-spatial IR observation

The high-spatial IR camera can provide additional spatial information to the high-speed IR camera. However, when interoperating the results, a rolling shutter effect (read the sensor signal line by line from top to bottom) must be considered^79^. For further illustration, an example (Trial #19) of high-spatial IR measurement is shown in Fig. 4c. The white line in frame *t* = 0.53–1.52 ms represents the laser starting because it is the first row of data with meaningful readings. Starting from then, in addition to the spatial increment, each row to the bottom direction has a 1/55 ms time increment because each image has 55 pixels in the vertical direction and a total span of 1 ms. The first line in frame *t* = 1.53–2.52 ms represents *t* = 1.90 ms (the laser off time); the second line represents *t* = 1.95 ms. The temperature profiles of these two lines are plotted in Fig. 4d, e, respectively. Additionally, the temperature profiles of the same location and timestamp from the high-speed IR have also been plotted in these two figures. The temperature readings from two IR cameras showed high consistency, and the high-spatial one provides more details. Since the laser has a Gaussian profile, the melt pool of the spot melting is generally radial symmetry (strong keyhole mode may have fluctuations, but this scenario should be avoided in the production). Therefore, a single line scan across the center of the melt pool might be a good choice in future research since a higher sensing frequency may be achieved.

### Uncertainties

This section aims to quantify the uncertainties from the measurement itself and the feature extraction process. As for the X-ray-related geometrical data, all solid–liquid boundaries of the melt pool in this work are manually labeled, and the noisy image can introduce uncertainties. The enlarged image in Fig. 3i shows that the boundary has a width of around 5 pixels. The location of the boundary measurement must have a minimum accuracy of ± 2 pixels, representing a ± 4 µm uncertainty. The 2 µm*/*pix spatial resolution can also have an uncertainty from the focus and the sensor chip manufacturing process. However, since this type of uncertainty is significantly less than ± 4 µm, it has been neglected. The boundary velocities (*V*_*D*_ and *V*_*W*_) uncertainty comes from two aspects, namely, dimension and time. The dimension uncertainty here is the same as mentioned above ( ± 4 µm). The timing uncertainty has been neglected because it is significantly smaller than the cooling time. To be conservative, Trial # 17 (Ti–6Al–4V, 130 W, 0.6 ms) is used for estimating the boundary speed uncertainty since it has the smallest energy density and shortest cooling time (0.2714 ms). Using the melt pool dimension uncertainty divided by the cooling time, the corresponding boundary speed uncertainty is ± 0.015 m*/s*.

IR measurement (*T*_*avg*_, *T*_*max,*_ and temperature profiles) uncertainties come from three perspectives: ambient condition, emissivity, and sensor response. The software has integrated features to correct the ambient temperature, and the reflections from the environment can be neglected because their temperatures are significantly lower than the melt pool. The emissivity calibration brings the most significant uncertainty because it cannot be accurately set (the smallest increment is 1%). Therefore ± 0.5% of this part should be accounted for, corresponding to ± 2.6% uncertainty of the temperature. The emissivity calibration method presented in "X-ray data pre-processing and dimensions calculation methods" Section is a relatively simple method since it assumes the emissivity as a constant. This is valid for the melt pool boundary region, and the number presented in this work exactly matched the one in the literature^57,59^.

As for hotter regions such as the keyhole, Qu et al.^80^ have reported that the emissivity could be as low as 0.04, resulting in a higher temperature reading. However, precisely interpreting the keyhole temperature data is not the scope of this paper. Even though the measured *T*_*max*_ has a lower temperature than reality, the trends within and across the different processing conditions are preserved. This could be used for data correlation and future control purposes. In addition to the emissivity-related uncertainty, both the high-speed and high-spatial IR cameras have sensor response uncertainties (the capability to distinguish different temperatures). They are 0.05 and 0.005 °C for high-spatial and high-speed IR cameras. They were neglected since their scale is relatively small compared with the Ti–6Al–4V and SS 410 melting temperature. The uncertainty of the thermal gradient *G*, and cooling rates (*C*_*avg*_, and *C*_*max*_) is negligible because they reflect the temperature trend and are only influenced by the abovementioned negligible sensor response uncertainty. Additionally, propagation of these uncertainties through averaging will further reduce the uncertainty.

Besides the abovementioned sources, the melt pool surface fluctuation might influence the temperature measurement. However, since this phenomenon is highly sophisticated, its quantification could not be solved by the experimental data and the analysis reported in this paper. It will be explored in our future work. As a result, the uncertainty analysis presented in this section is the lower bound of the actual uncertainty.

## Correlation analysis and IR-based melt pool information prediction capability

This section conducts the correlation analysis between the X-ray data and IR data. Section "Keyhole prediction" first demonstrates the predictions of the keyhole based on IR observations. Section "Melt pool shape prediction" then estimates the correlation between the melt pool dimensions and the high-speed IR readings, indicating IR observation can be used for melt pool shape prediction. Section "Thermal gradient and solid–liquid boundary velocity prediction" discusses the thermal gradient and boundary velocity prediction, which are important enablers for future microstructure control.

### Keyhole prediction

**Figure 5:**
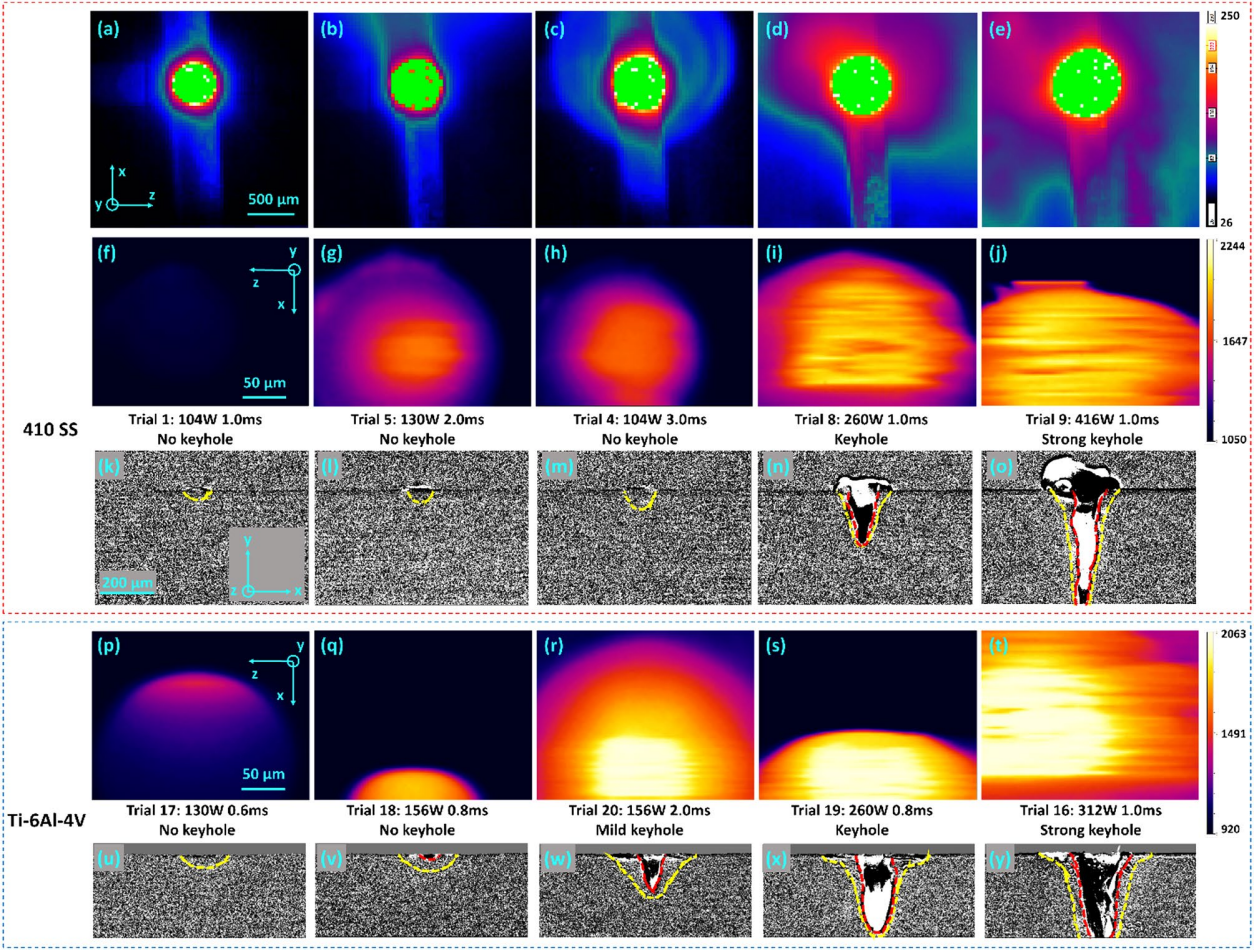
(**a**–**e**) The high-speed IR observations of different processing conditions at the frame right before the laser shuts off. The last two (**d** and **e**) have strong vapor plumes indicating a keyhole. (**f**–**j** and **p**–**t**) The high-spatial IR observations of different processing conditions at the frame right before the laser shuts off where i, j, r, s, t have fringe patterns, which indicate a keyhole. (**k**–**o** and **u**–**y**) The corresponding x-ray images right before the laser shuts off, where yellow dashes indicate the solid–liquid boundary and red dashes indicate the liquid–gas boundary. (Temperatures all in °C).

By comparing the IR frames right before the laser turns off with the X-ray imaging in different trials (Fig. 5), both IR cameras were able to identify the occurrence and the level of the keyhole, which is known to correlate with the porosity. For the high-speed IR with a low-temperature filter, the large purple region represents a strong vapor plume, an effective keyhole indicator (Fig. 5d, e compared with 5a–c). Due to the relatively low temporal resolution, the high-spatial IR shows interesting fringe patterns in the image when the keyhole forms (Fig. 5i, j, r, s, t). The fringe patterns result from the rolling shutter effect^79^, which means the fast-moving object is distorted by a relatively slow-rolling shutter camera (the camera with a sensor reading line by line instead of reading all pixels at once). When the keyhole occurs, the melt pool's surface oscillates, and the ripples may reflect unblocked laser spectra to the IR camera. These factors make the high-spatial IR show fringe patterns. This phenomenon is a keyhole indicator in both 410 SS and Ti–6Al–4V trials. The high-speed camera can be mounted onto the co-axial position to achieve laser tracing in an industrial setup.

By comparing the thermal images shown in Fig. 5, the level of the keyhole is more related to the laser power itself than the energy input. For example, Trial #5 and #8 have the same laser energy input (laser power times dwell time), but Trial #8 has a more severe keyhole due to its higher laser power. Similarly, Trial #19 has an even lower energy input than Trial # 20; however, the broader fringe pattern in Fig. 5n indicates a stronger keyhole due to the higher power setting. This finding shows that L-PBF printing with a random spot scan strategy cannot infinitely speed up by shortening the dwell time and increasing the power to compensate for the energy density. This is because a strong keyhole will occur, resulting in significant porosities.

By comparing the 410 SS cases with Ti–6Al–4V cases in Fig. 5, 410 SS is harder to melt by the 1070 nm laser. This is because Ti–6Al–4V has a higher absorptivity at 1070 nm (60%) compared with steel (30%)^74,81^. Specifically, Trial #5 (410 SS, 130 W, 2 ms) has the same laser power as Trial #17 (Ti–6Al–4V, 130 W, 0.6 ms). Even though Trial #5 has a three-times longer melting time, the melt pool size is smaller. Similar observations have been found by comparing Trial #8 with #19 and Trial #9 with #16. Higher thermal conductivity of the 410 SS (15 W*/*m K compared with *7* W/m K) may also contribute to this^72^. The X-ray images in Fig. 5 show that the melt pool dimension ratios (W/D) of 410 SS trials are generally smaller than Ti–6Al–4V. This could be due to the Marangoni effect, which defines the melt pool's internal circulation direction^82^. The dimension ratio difference can infer that 410 SS printing should have a smaller hatch spacing when melting with the same layer thickness as Ti–6Al–4V. Moreover, since the keyhole in 410 SS cases is narrower, it is more likely to trap bubbles to form porosity. Therefore, printing 410 SS with a thicker layer height (over 100 µm) for a printing speed improvement is not recommended.

### Melt pool shape prediction

**Figure 6:**
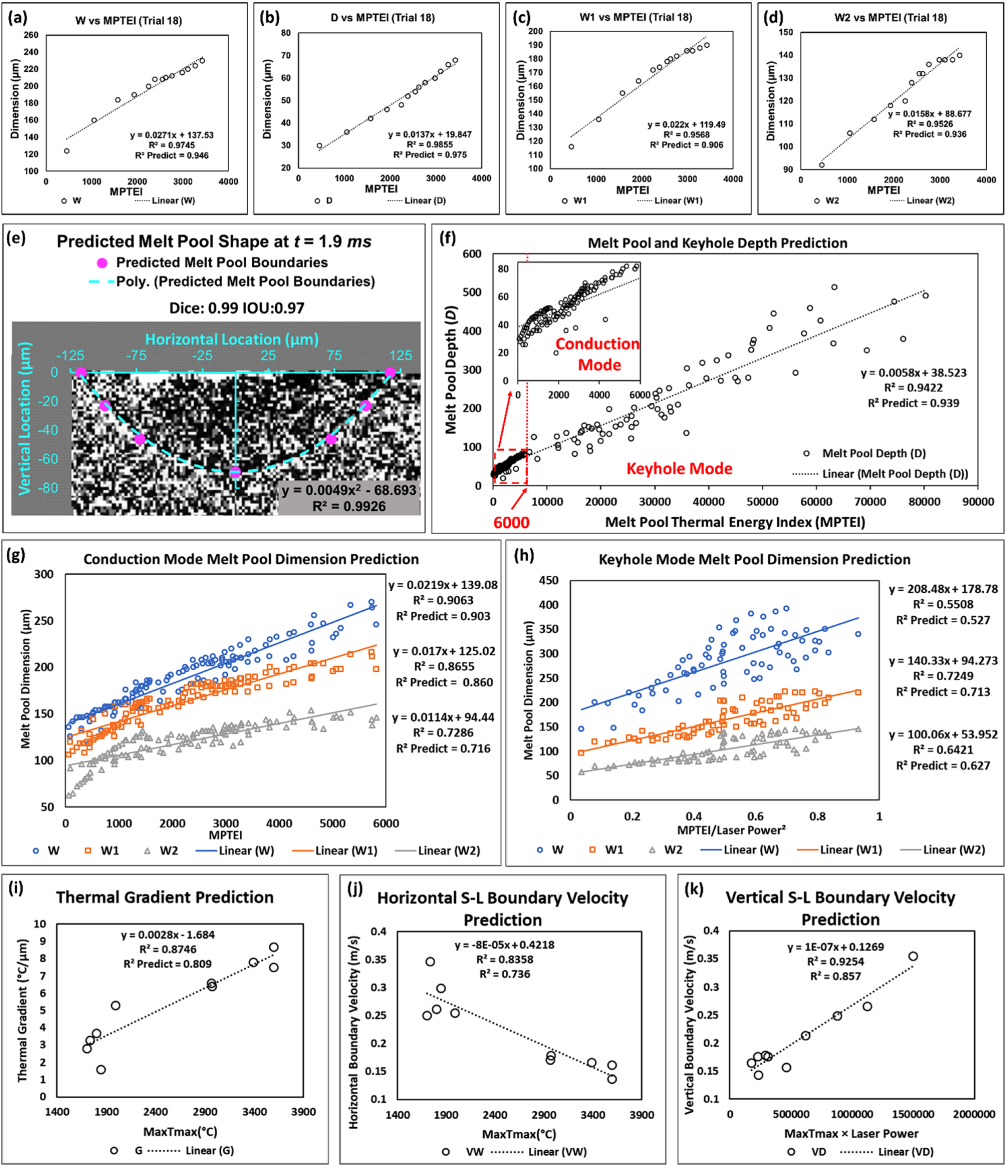
(**a**–**d**) The relationship between the melt pool dimensions and *MPTEI*, (Trial #18, Ti–6Al–4V, 156 W, 0.8 ms dwell time). (**e**) The predicted melt pool boundary based on the fitted equations at *t* = 1.9 ms of Trial #18. (**f**) Melt pool depth prediction based on *MPTEI*. Notice the melt pool depth is the same as the keyhole depth during keyhole mode. This plot includes all Ti–6Al–4V cases. (**g**) The correlation between *W*, *W*_1_, and *W*_2_ with *MPTEI* for all Ti–6Al–4V cases during conduction mode. (**h**) The correlation between *W*, *W*_1_, and *W*_2_ with *MPTEI* for all Ti–6Al–4V cases during keyhole mode. (**i**) The correlation between thermal gradient and *MaxTmax* for all Ti–6Al–4V cases. (**j**) The correlation between horizontal solid–liquid boundary velocity (*VW*) and *MaxTmax* for all Ti–6Al–4V cases. (**k**) The correlation between vertical solid–liquid boundary velocity (*VD*) and *MaxTmax*· *Laser Power* for all Ti–6Al–4V cases.

Strong correlations were found by plotting the melt pool dimensions (calculation method described in "X-ray data pre-processing and dimensions calculation methods" Section) against the *MPTEI* obtained from the high-speed IR. Examples of the melt pool dimension plots and dimension ratio plots against *MPTEI* are shown in Fig. 6a–d (Trial #18). During the melting process, the melt pool dimensions (*W*, *D*, *W*_1_, and *W*_2_) generally follow linear trends with *MPTEI*. The fitted equations are shown in the plots, and the high *R*^2^ and *R*^2^-predicted values indicate they are adequately fitted and have strong capabilities to predict new observations. Similar correlations were observed in all cases. The plots of all the Ti–6Al–4V trials are provided in Appendix B. Note that the fitted data only contains the steady melting or the keyhole melting part of the process because those are when the melt pool is truly developing.

In Appendix B9, Trial #20 (Ti–6Al–4V, 156 W, 2 ms) demonstrates a transition between conduction and keyhole mode. The transition happens when the *MPTEI* is around 6000, and the corresponding *T*_*max*_ value is around 2015 °C. When using 6000 as a threshold, the rest of the trials in Appendix B can be separated into two groups. Trials #11, 14, 15, 17, and 18 remain in conduction mode for the entire melting due to relatively low laser power, and the maximum *MPTEI* is all below 6000. In contrast, Trials #12, 13, 16, and 19 enter keyhole mode quickly and maintain a high *MPTEI* level (above 6000). The cooling interval is omitted in Appendix B9 because the high-speed IR camera cannot capture meaningful data when the melt pool temperature is below the minimum measuring temperature.

The melt pool geometry evolution can only be observed by high-speed X-ray imaging, a costly solution. The correlations shown in this section enable the prediction of melt pool dimensions based on high-speed IR camera data (*MPTEI*). Figure 6e demonstrates a predicted melt pool based on the fitted equations displayed in Fig. 6a–d. This is accomplished by calculating the predicted *W*, *W*_1_, *W*_2_, and *D* based on the *MPTEI* reading, then plotting them and fitting a second-order curve as the melt pool boundary. As Fig. 6e shows, the predicted melt pool nicely matched the X-ray observation. This fitted melt pool is for *t* = 1.9 ms, which is not accounted for in the correlation fittings. The predicted melt pool follows a second-order polynomial curve, and *R*^2^ is also presented in the figure. The prediction accuracy is quantified by the Dice Coefficient and Intersection Over Union (IOU), two commonly used mask prediction quality measurements^83^. These two scores are very close to one, which indicates a strong correlation, and they are both shown in Fig. 6e.

When plotting the dimension terms (*D*, *W*, *W*_1_, and *W*_2_) of all the trials against the *MPTEI*, strong linear correlations have also been observed. Figure 6f shows that the melt pool depth (*D*) can be nicely predicted by *MPTEI* regardless of processing conditions. As discussed in the first paragraph of this section, the data points can be separated into conduction and keyhole mode using the 6000 *MPTEI* threshold. Data points during conduction closely follow a linear trend and show little scatter from the trend due to the stability of the melt pool. In contrast, the data points are more sporadic for the keyhole mode since the melt pool is volatile. The correlation developed in this figure also indicates the high temporal resolution keyhole depth prediction can be achieved because the keyhole depth is generally the same as the melt pool depth during the keyhole mode. As Appendices B9 shows, *W*, *W*_1_, and *W*_2_ do not follow the same linear trend during the conduction and keyhole mode. Therefore, they are analyzed separately.

Figure 6g plots *W*, *W*_1_, and *W*_2_ data during the conduction mode against *MPTEI* for all cases. Nice linear correlations have been discovered for the three dimension terms. Similarly, Fig. 6h shows the keyhole mode *W*, *W*_1_, and *W*_2_ observations. Instead of directly using *MPTEI*, this plot uses *MPTEI*/*laser power*^2^ for compensating the influence of high laser power. This is because the higher the laser power is, the lower the width to depth ratio will be, resulting in relatively small *W*, *W*_1_, and *W*_2_ when surface thermal radiation is high. By comparing R^2^ and R^2^ predict values between the conduction and keyhole modes (see Fig. 6g, h), three melt pool width terms are harder to predict precisely during the keyhole mode. However, a strong keyhole should be generally avoided during the L-PBF process since it is prone to leave trapped gas porosity.

### Thermal gradient and solid–liquid boundary velocity prediction

As introduced in "Introduction" Section, thermal gradient and solid–liquid boundary velocity are two instrumental pieces of information for understanding the fundamentals of L-PBF, knowing they are strongly correlated with microstructure. The extracted term *MaxT*_*max*_ has a strong capability to predict them. The correlation plots for thermal gradient and *MaxT*_*max*_ is shown in Fig. 6i. The positive linear correlation means cases with higher laser power will have higher thermal gradients. Figure 6j plots the relationship between *V*_*W*_ and *MaxT*_*max*_. The negative trend indicates that the melt pool shrinks at a higher speed during cooling for cases with lower laser power. This is because the melt pool shape is flat when laser power is low. The relationship between *V*_*D*_ and thermal data has also been investigated. Instead of directly using *MaxT*_*max*_, this thermal feature has been scaled by the laser power because high laser power will result in a narrow and deep melt pool that shrinks faster. The correlation of *V*_*D*_ and *MaxT*_*max*_ ·*laser power* is shown in Fig. 6k. The positive trend confirms that a higher *V*_*D*_ will be expected when the laser power is high.

## Conclusions

This work explores the feasibility of using multiple synchronized sensors to observe the melt pool dynamics of L-PBF. Specifically, the high-speed X-ray can reveal melt pool geometrical information. The melt pool dimensions, solid–liquid boundary velocities, and keyhole information can be extracted. When equipped with a high-temperature filter, the high-speed IR thermal camera can provide dynamic thermal profiles of the melt pool. Thermal terms such as maximum temperature, thermal gradients, and cooling rates can be quantified. Using the low-temperature filter, the high-speed IR camera can observe the dynamics of vapor plumes. This can help understand the temperature and dynamics of vapor during printing. The customized high-spatial IR camera in this study currently has the highest spatial resolution (3.6 µm) in the literature. It provides additional spatial information to the high-speed IR camera. By analyzing the data, both IR cameras can predict the occurrence and the level of keyholes. Strong correlations between the X-ray and high-speed IR data are found. High-speed IR readings could infer melt pool shapes and keyhole information based on those correlations. This finding means the high-speed IR, which is a cheaper solution, can potentially measure the data that currently can only be obtained by the expensive high-speed X-ray. Since the high-spatial IR can reveal the severity of the keyhole as well, it has a great potential to be used for process monitoring and quality control in L-PBF. Additionally, the analysis shows that the high-speed IR data can be used for estimating the thermal gradient and solid–liquid boundary velocities, which enables future microstructure control during the printing.

## Supplementary Information

Supplementary Information.

## Data Availability

The raw/processed data required to reproduce these findings cannot be shared at this time due to technical or time limitations. They will be made available before eventual acceptance and publication. The point of contact for future correspondence to this statement: Zhenyu Kong (zkong@vt.edu).
