# Supplementary material for: In situ melt pool measurements for laser powder bed fusion using multi sensing and correlation analysis
Source: Sci Rep. 2022 Aug 12;12:13716. doi: 10.1038/s41598-022-18096-w (PMC9374674; doi:10.1038/s41598-022-18096-w)

Appendix

Appendix A1. High-speed IR window transmission spectrum from Kurt J. Lesker Company


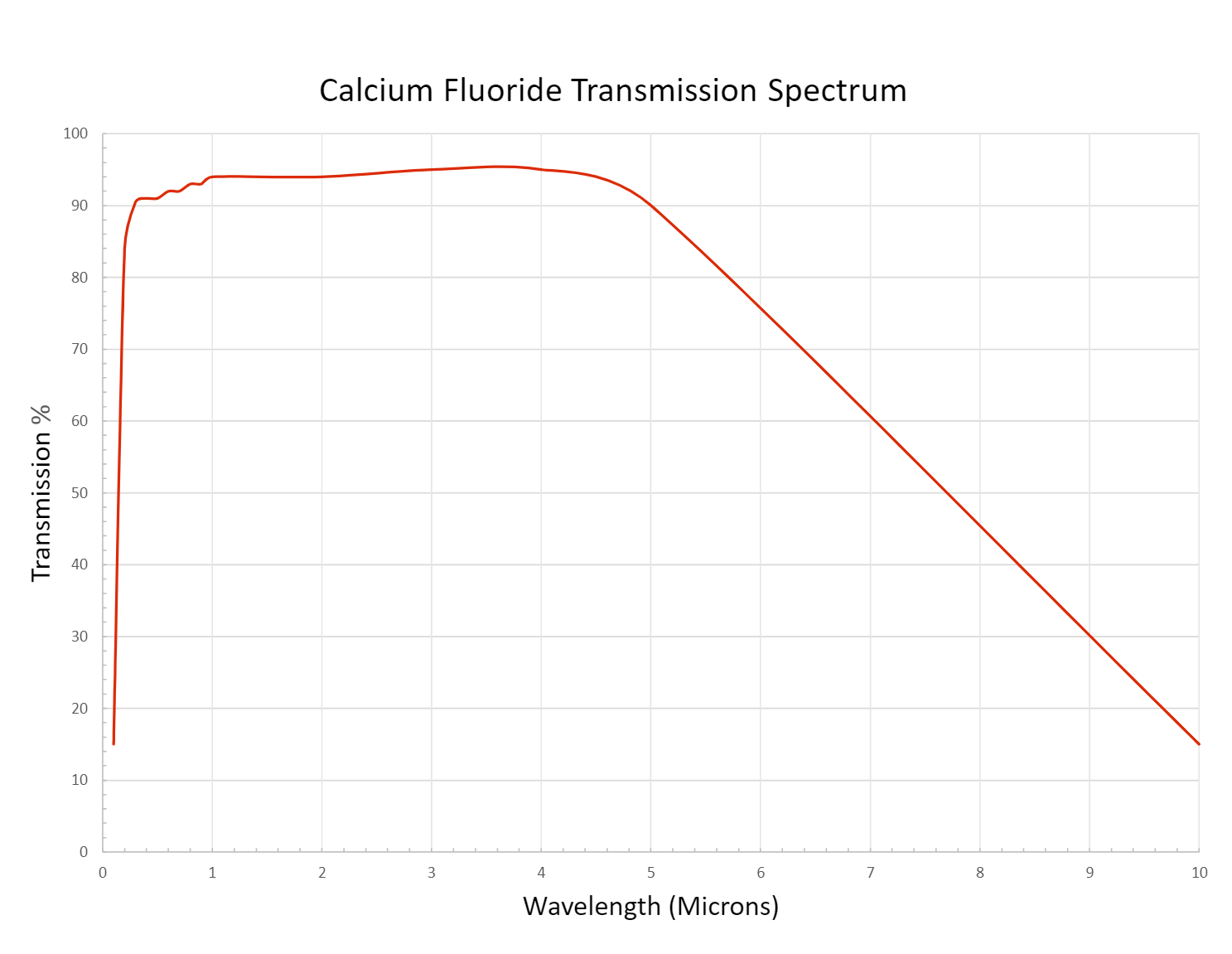


Appendix A2. High-spatial IR filter coating transmission spectrum from Edmund Optics
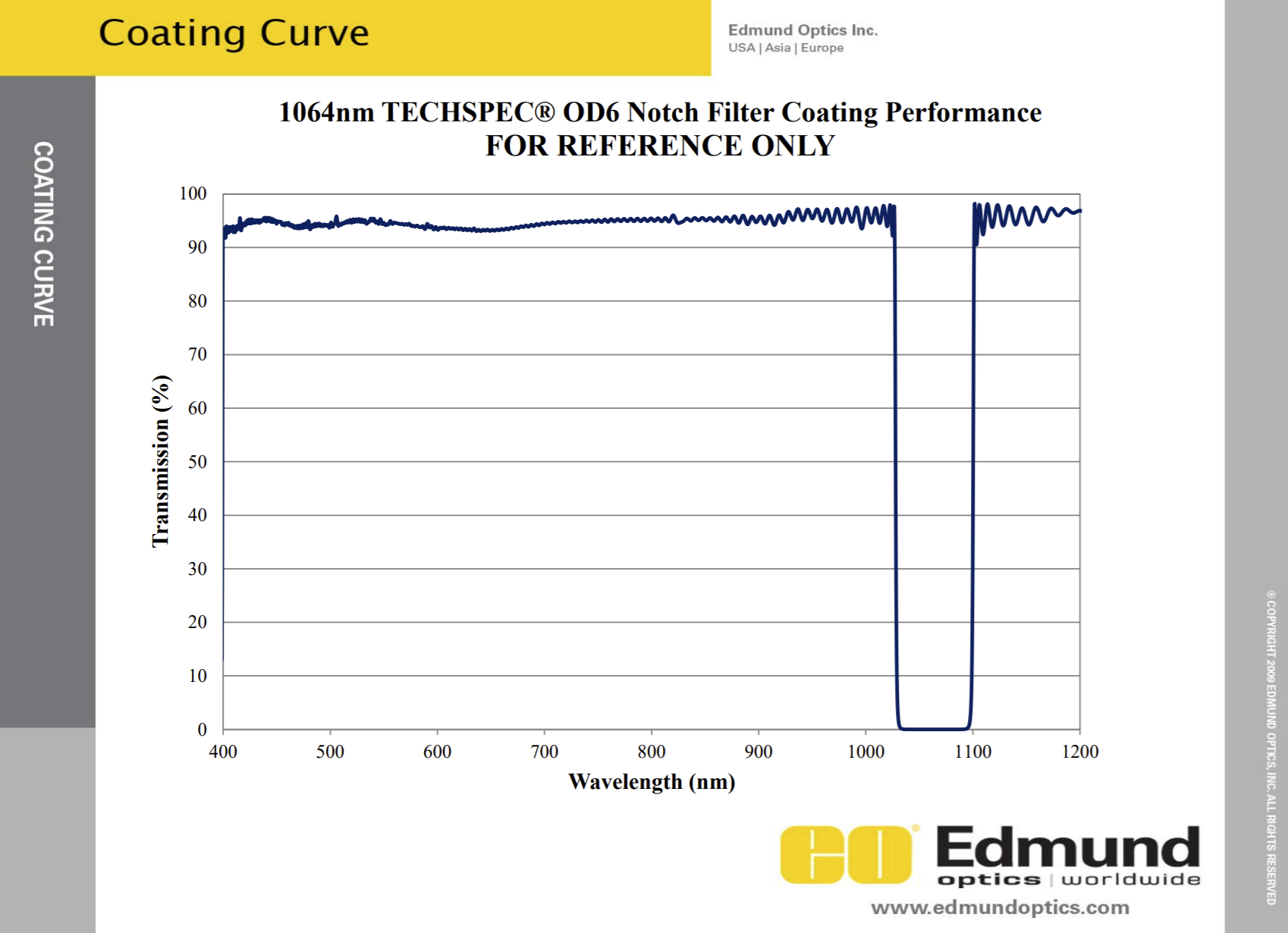


Appendix A3. Measured chemical composition (in wt.%, with Ti in balance) of Ti-6Al-4V plate procured from TMS Titanium.

| **Composition (wt.%)** | | | | |
| --- | --- | --- | --- | --- |
| Al | V | Fe | O | C + H + N + Y |
| 6.11 | 3.98 | 0.2 | 0.16 | < 0.02 |

Appendix A4**.** Measured chemical composition (in wt.%, with Fe in balance) of 410 SS produced using laser wire-fed DED.

| **Composition (wt.%)** | | | | | | | |  |
| --- | --- | --- | --- | --- | --- | --- | --- | --- |
| C | Cr | Ni | Mo | Mn | Si | P | S | Cu |
| 0.11 | 12.5 | 0.1 | 0.03 | 0.45 | 0.39 | 0.01 | 0.01 | 0.14 |

Appendix B1. (a-d) The relationship between the melt pool dimensions and MPTEI. (Trial #11)


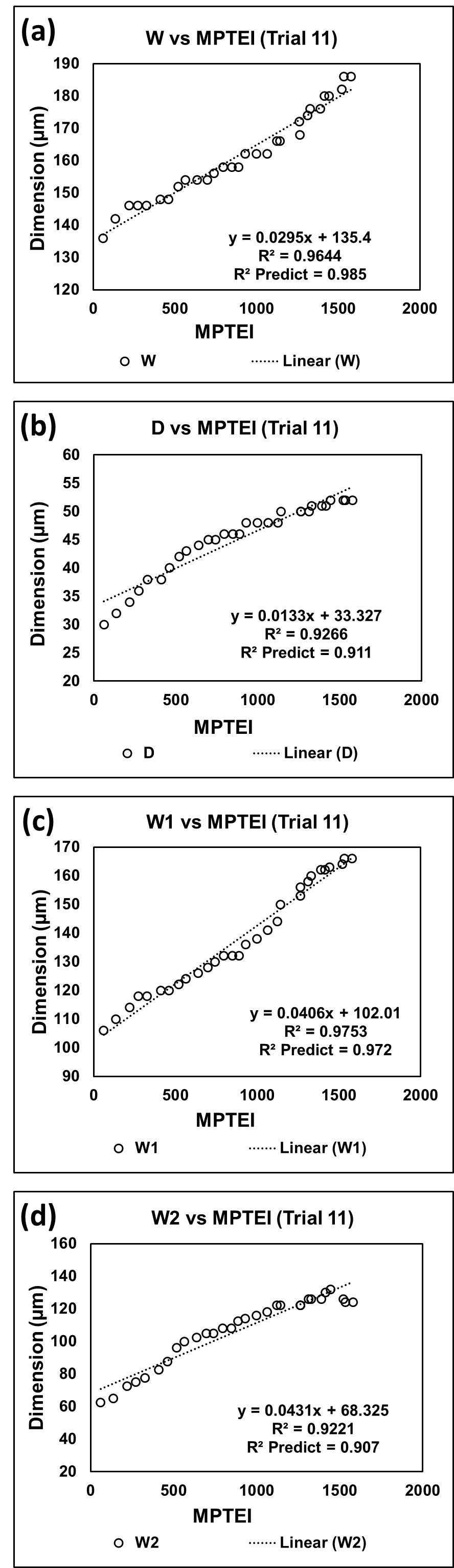


Appendix B2. (a-d) The relationship between the melt pool dimensions and MPTEI. (Trial #12)


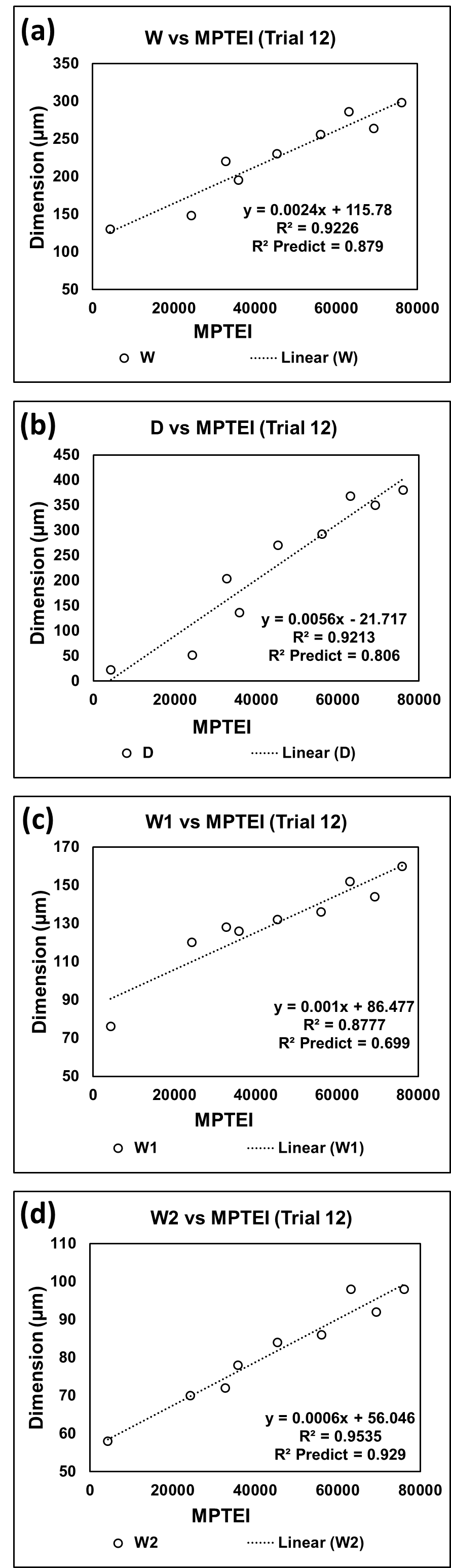


Appendix B3. (a-d) The relationship between the melt pool dimensions and MPTEI. (Trial #13)


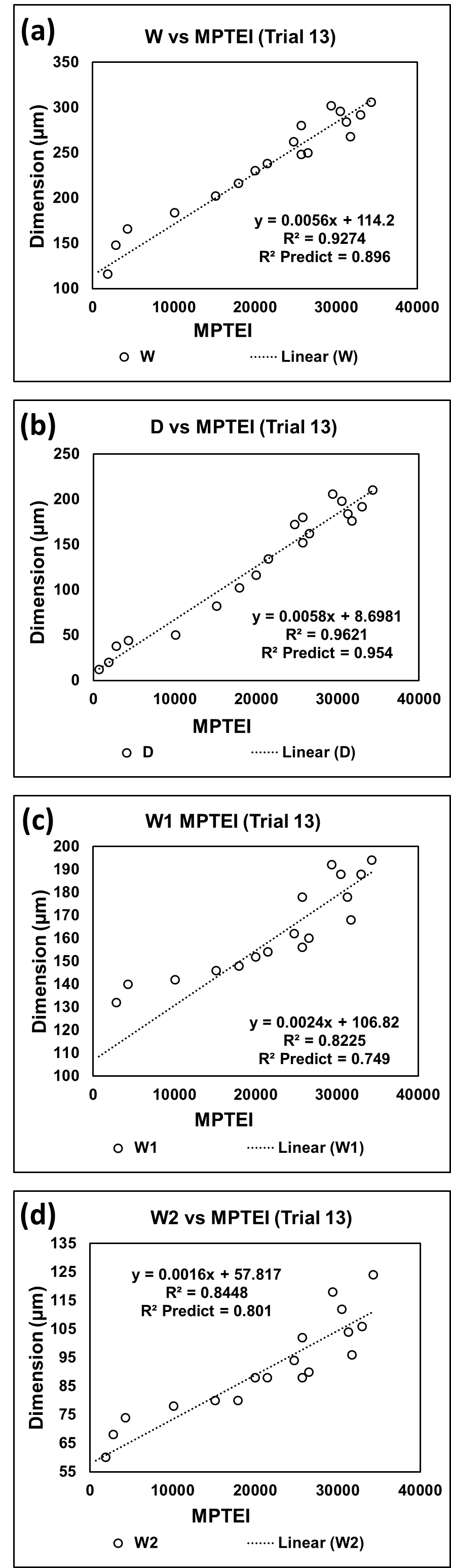


Appendix B4. (a-d) The relationship between the melt pool dimensions and MPTEI. (Trial #14)


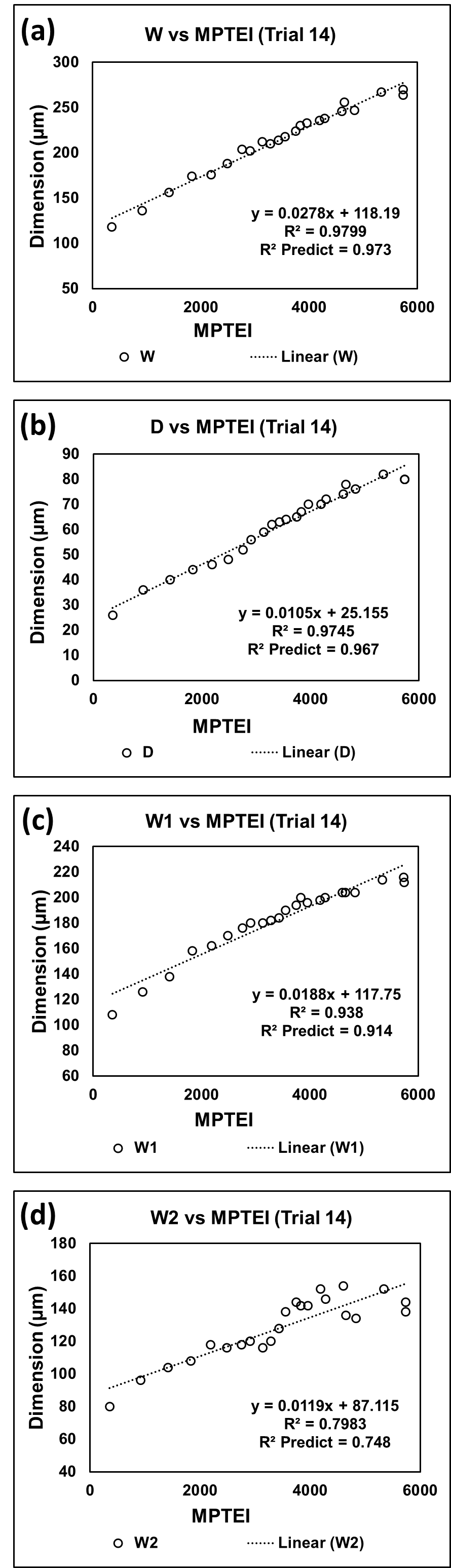


Appendix B5. (a-d) The relationship between the melt pool dimensions and MPTEI. (Trial #15)


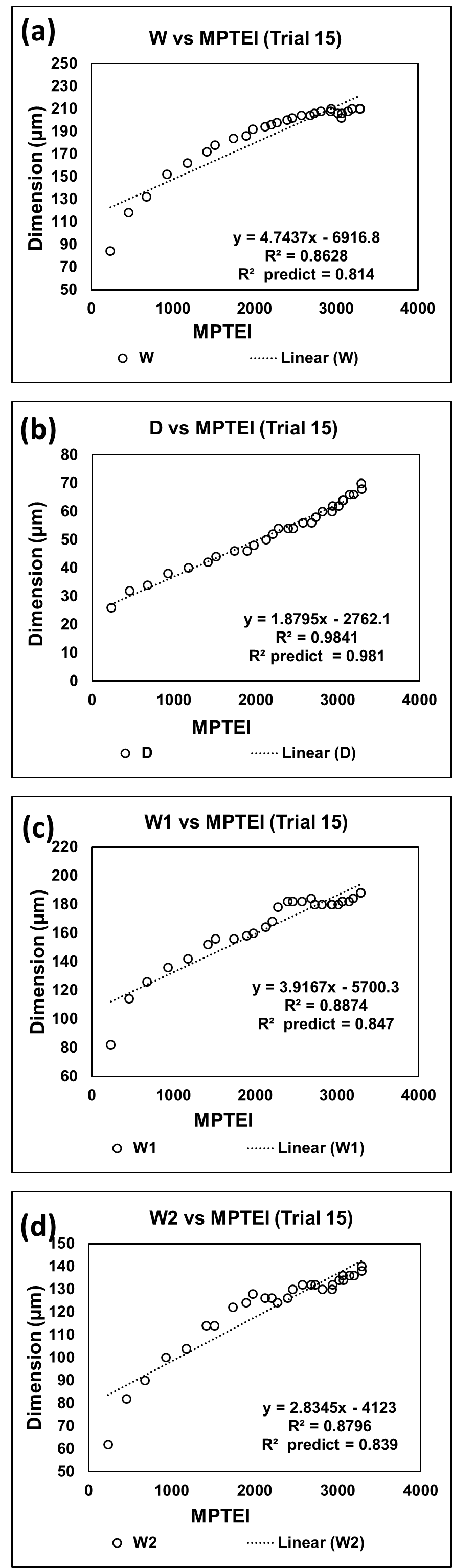


Appendix B6. (a-d) The relationship between the melt pool dimensions and MPTEI. (Trial #16)


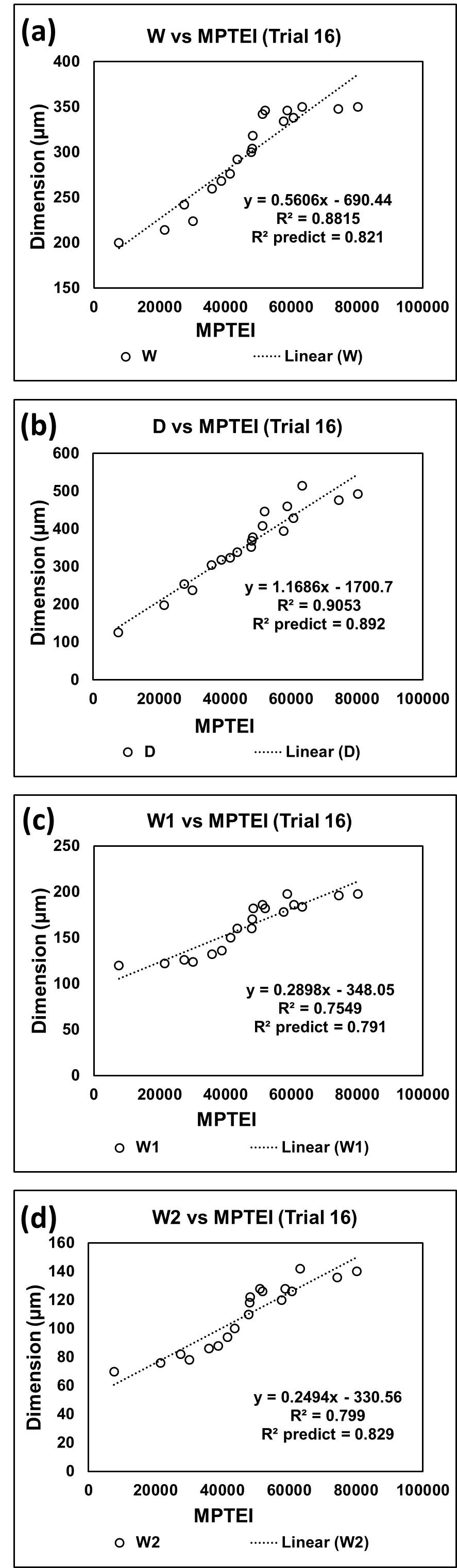


Appendix B7. (a-d) The relationship between the melt pool dimensions and MPTEI. (Trial #17)


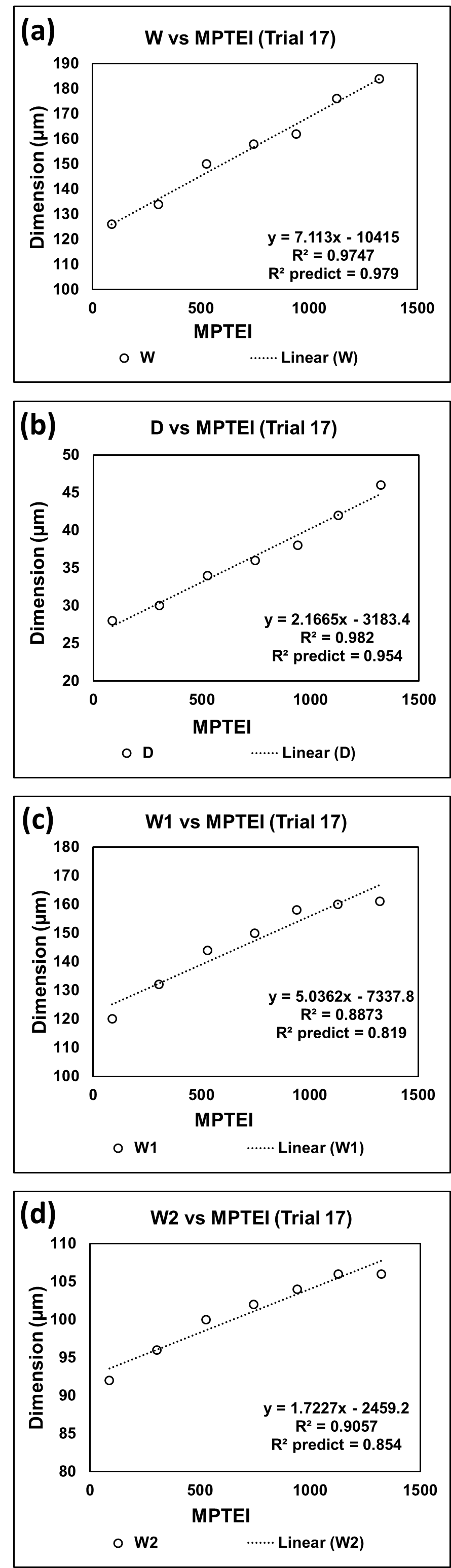


Appendix B8. (a-d) The relationship between the melt pool dimensions and MPTEI. (Trial #19)


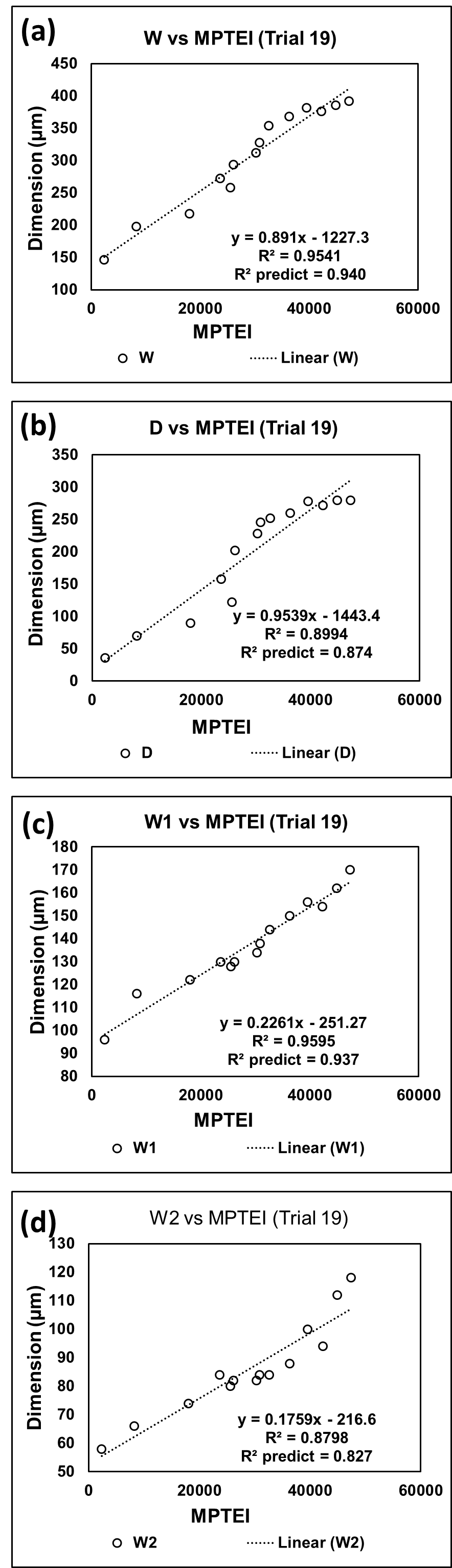


Appendix B9. (a-d) The relationship between the melt pool dimensions and MPTEI. (Trial #20) Note conduction mode and keyhole mode are fitted separately.


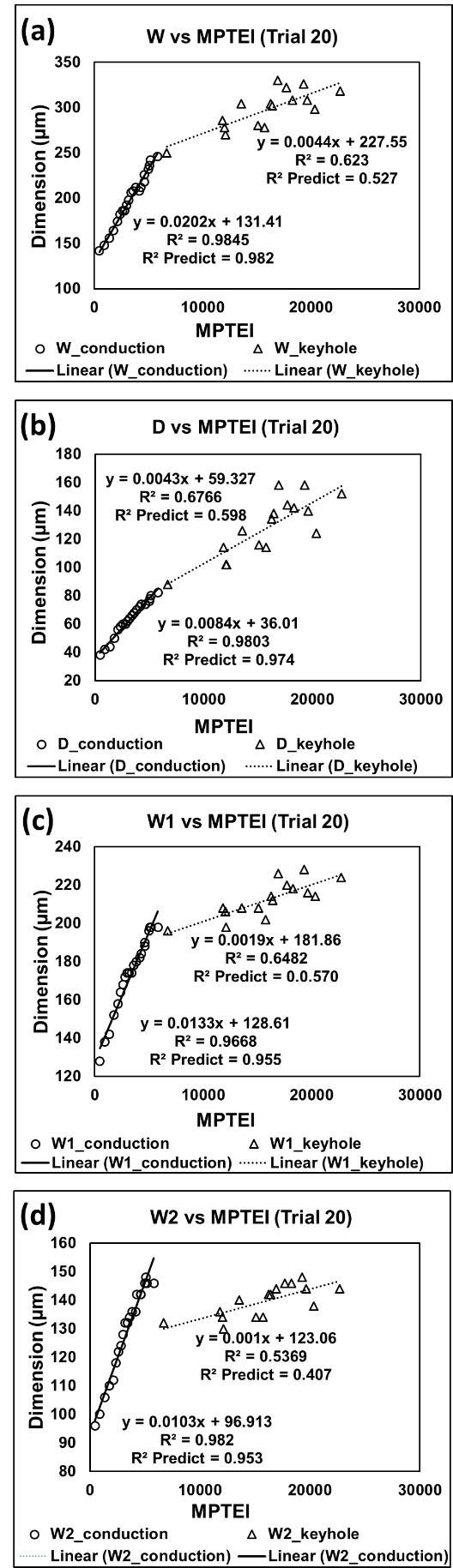

Supplement: Supplementary file 1 — Supplementary Information. [file 41598_2022_18096_MOESM1_ESM.docx]
